# A framework to refocus the conversation around the welfare of UK purebred cats

**DOI:** 10.1017/awf.2025.10051

**Published:** 2025-11-28

**Authors:** Claire Roberts, Rae Foreman-Worsley, Dan G. O’Neill, Jennifer L. McDonald

**Affiliations:** 1 Feline Welfare and Operations Directorate, Cats Protection, UK; 2 The Royal Veterinary College Department of Pathobiology and Population Sciences, UK

**Keywords:** Animal welfare, breeding, cats, extreme conformation, pedigree, purebred

## Abstract

Previously, consideration of purebred cat welfare has focused heavily on links between specific breeds and their predispositions to disease, extreme morphology, and behavioural traits. While these are certainly important to consider, negative messaging about purebred cats is often poorly evidenced and can be alienating to owners and breeders, with consequent limited or even negative welfare gain. Negative focus on individual breeds also risks overlooking the wider picture, where propagation of genetic traits that impinge upon welfare should be avoided across all cats (*Felis catus*). An account of purebred cat welfare must also consider husbandry and breeding practices, new experimental breeds and motivations behind changing acquisition trends. This review used a framework based on the five welfare needs from UK legislation, bolstered by published feline quality of life assessment tools, to review the literature on purebred cats. This aimed to re-orient the discussion on purebred cat welfare away from excessive focus on individual breed statements and instead explore broader, generalisable evidence-based welfare considerations. The review concludes that purebred cat welfare in the UK falls short of ideal in numerous ways. These include more obvious conformational concerns, such as osteochondrodysplasia in Scottish Folds and the increasingly flattened faces of brachycephalic cats. Several areas where strong evidence is lacking were also identified, including current breeding conditions, impact of morphological changes on social behaviour, and the breeding and keeping of hybrid cats. More evidence on the motivations behind owning cats with specific morphology is also required to better target interventions to improve the lives of cats.

## Introduction

Domestic cats (*Felis catus*) have been integral to human society for around 10,000 years (Vigne *et al.* 2004). Cats originally co-habited alongside humans in settlements as a traditionally useful means for pest control due to the abundance of prey attracted to food stores; cats were not selectively bred to fulfil this working role (Serpell 2000). Genetic data suggest that these generic types of cats spread globally before most of the currently extant breeds were created, indicating that current domestic short, medium and long hair cats are not mixed-breed descendants of different purebred breeds, in contrast to current ‘mongrel’ dogs (Lipinski *et al.* 2008; McGrath *et al.* 2021). In fact, records show that most of the current distinct cat breeds were only invented within the last 70 years (Wastlhuber 1991, cited in Lipinski *et al.* 2008).

**Figure 1.**
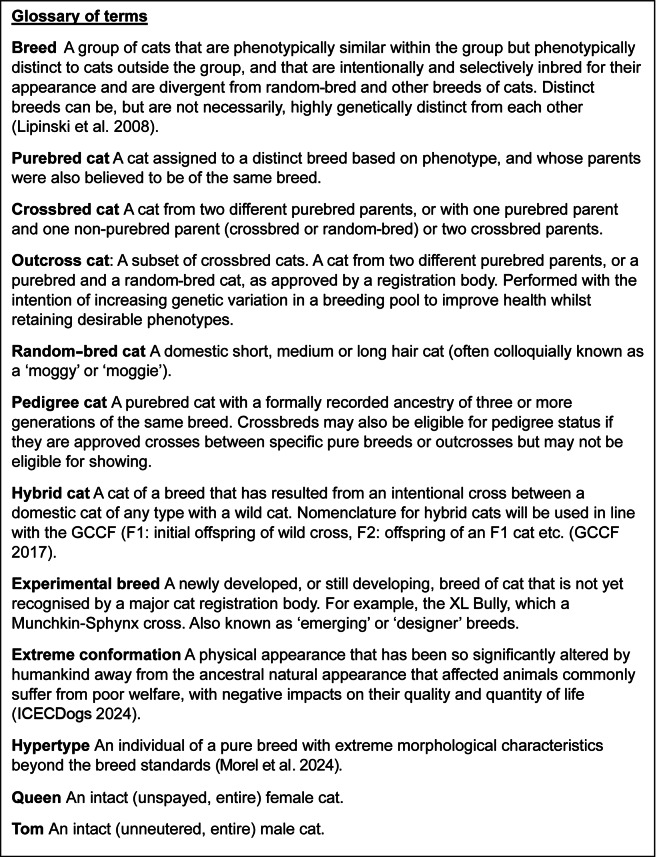
Glossary of terms used in this Horizon Topic paper regarding the welfare of UK purebred cats.

**Figure 2.**
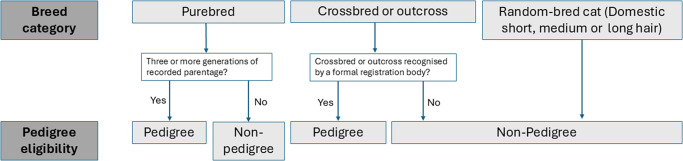
Eligibility to obtain pedigree status for purebred, crossbred and random-bred cats. Cats from all three categories can be registered with certain breed organisations (depending on the organisations’ guidelines).

‘Pedigree’ and ‘purebred’ as terms are often used interchangeably in the literature, so it is important to define these terms as used in the current review, along with some others. The definitions used throughout this Horizon Topic are shown in the glossary of terms (Figure 1) with the relationship between the category of breeds and eligibility for pedigree status shown in Figure 2. Purebred, crossbred and random-bred cats can variably be registered with cat registration bodies, depending on the rules of that organisation (e.g. The International Cat Association [TICA] undated a, b). It is important to note that as crossbred cats can be descended from purebred cats, some of the welfare issues discussed throughout this review may also apply to certain groups of crossbred cats.

There is no definitive list or number of recognised cat breeds in the UK, as formalisation of a breed status requires recognition from a registration body of which there are three in the UK. At the time of writing, the Governing Council of the Cat Fancy (GCCF) has 39 fully recognised breeds (GCCF undated a), Felis Britannica (the UK arm of Federation Internationale Feline [FIFe]) has 48 fully recognised breeds (FIFe undated) and TICA has 73 fully recognised breeds (TICA undated a). Some breeds are recognised by more than one registration body, with 30 breeds fully recognised in the UK by all three registration bodies (Table S1; Supplementary material). Table S1 lists these UK-recognised breeds alongside newer experimental breeds that are in the preliminary stages of being recognised. The difference in number of recognised breeds between these organisations stems in part from FIFe and TICA listing short- and long-haired variants of breeds separately, but also from reluctance by the GCCF to accept breeds with known welfare or ethical concerns (GCCF undated b). The proportions of purebred and crossbred cats in the UK that have a pedigree is unclear. Although 18,535 cats were registered in the GCCF in 2024 (GCCF 2024), there are no available data as to how many cats from the UK are registered with TICA and FIFe. In one report, 46% owners of cats that were ‘non-moggies’ reported having paperwork to confirm the breed of their cat (Cats Protection 2024). There are also unknown numbers of experimental and/or rare cat breeds that are not yet recognised by any registration organisation. Tables S2 and S3 (Supplementary material) list hybrid and non-hybrid breeds, respectively, that are known to the authors but not currently accepted by any UK registration body.

In the UK, purebred cats have traditionally comprised a small proportion of all owned cats. The most recent estimates from veterinary surveillance studies reported that 8.3–10.4% of cats attending UK veterinary practices were purebred (O’Neill *et al.* 2015; Sánchez-Vizcaíno *et al.* 2017). Figures from a survey of over 6,300 cat owners indicate 30% of UK cats in 2024 were reported as pedigree or purebred (Cats Protection 2024). Although this is a drastic increase, these figures have very different methods of collection and are not directly comparable. The veterinary surveillance studies include only cats registered with a participating veterinary practice so may be subject to bias if purebred cats are more or less likely to be registered and also assumes that cat breeds are accurately recorded. The survey relies upon owner reporting of the breed of their cat and may also be subject to selection bias on those owners who choose to respond to the survey. In the same 2024 survey, more purebred cats were acquired in the last 12 months than random-bred cats which does indicate that this proportion is rising (Cats Protection 2024).

This proportion of purebred cats is still lower than for dogs, where 69.4 to 84.1% of dogs from veterinary surveillance studies are reported as purebred (Sánchez-Vizcaíno *et al.* 2017; O’Neill *et al.* 2023b). However, desirability and accessibility of purebred cats (compared with random-bred cats) has the potential to continue to increase with ongoing global visual media and communication networks, which seem likely to drive trends and facilitate the remote purchasing of kittens from breeders. This is a worry given the highly concerning welfare issues associated with some purebred cat traits, breeding practices and husbandry that will be discussed below.

Health and welfare impacts on cats from their breed assignment can be considered under three broad domains: breed predisposition to certain diseases; breeds with extreme conformations; and breed-associated behavioural issues (Sandøe *et al.* 2015). In addition, loss of genetic diversity following heavy inbreeding with artificial selection to establish individual breeds can lead to small gene pools and ‘inbreeding depression’, which can promote disposition to disease and fertility issues (Casal 2022). While there is a growing body of literature investigating the impact of cat breed on welfare, the evidence base for breed-related issues in cats remains weak and lags far behind that for dogs. Methodological variation between the published feline studies that include case-control (Vapalahti *et al.* 2024) and cohort studies (Granström *et al.* 2011), data from national veterinary databases (O’Neill *et al.* 2015), individual practices (Lederer *et al.* 2009), owner-reported surveys (Salonen *et al.* 2019) and insurance data (Egenvall *et al.* 2010; Öhlund *et al.* 2015; Ström Holst *et al.* 2017) also make comparison between studies and results difficult. Many studies are severely underpowered, with sample sizes for individual breeds often insufficiently large for robust statistical inference and often forcing researchers to group multiple individual cat breeds as a single purebred monolith that may miss nuances between breeds (e.g. De Santis-Kerr *et al.* 2006; Finch *et al.* 2016; Köhler *et al.* 2016). The consequent diminished holistic understanding of the health and welfare of individual cat breeds limits the applied relevance of findings, with general statements regarding purebred welfare likely to be inaccurate when later applied to individual breeds, an ecological fallacy (Winzar 2015).

An added complication is residual variability of conformation and behaviours between cats within individual breeds, such as hypertypes (Figure 1). This results in individual cats within the same breed having differing degrees of welfare compromise, and risking blanket statements regarding breed health and welfare alienating those owners and breeders within these breeds who do prioritise non-extreme conformations. Finally, the large number of extant breeds and the emergence of new breeds mean the published evidence from breed-specific studies often lags behind trends. The full extent of the welfare concerns surrounding purebred cats is therefore difficult to assess but nonetheless must be addressed if the domestic cat species is to be protected from a replication of the purebred crisis that is currently engulfing the domestic dog species (Brachycephalic Working Group [BWG] 2024; ICECDogs 2024; LAGECDogs 2024).

Approaches currently being used to examine breed health in dogs offer an opportunity for similar approaches in cats. In dogs, concerns about breed health have been widely discussed for over 60 years. Despite this, even determined focus during subsequent decades on breed health reform has resulted in very limited progress towards reducing ownership of extreme breeds or in moving the conformation of dogs within extreme breeds substantially towards more moderate conformation (Hodgman 1963; O’Neill *et al.* 2023b). Learning from these failures over the past decade, welfare research and advocacy has increasingly moved away from focusing welfare judgements on specific breeds, and instead towards assessment of the welfare impacts from the harmful physical features themselves, with subsequent consideration on how these can be applied to affected breeds. This approach appears to have gained more traction whereby individuals and organisations who are highly wedded to supporting certain breeds often remain open to accepting that there are welfare concerns related to the key extreme conformations within these breeds. For example, the BWG has supported the innate health concept that specifies the functions that a physically healthy dog should be capable of undertaking (e.g. breathing and blinking) and subsequently identifies conformations that typically preclude these natural functions (e.g brachycephaly and bulging eyeballs) (UK Brachycephalic Working Group 2022). Building on this learning, a similar framework to identify the key requirements for a good life for a cat could be derived from the Animal Welfare Acts across the UK, which all list the five welfare needs that animal keepers should legally provide (Table 1): the needs for a suitable environment; a suitable diet; to be able to exhibit normal behaviour patterns; to be housed with or apart from other animals; and to be protected from pain, suffering, injury and disease.

**Table 1.** A framework for exploring the welfare of cats based on the five welfare needs for animals under UK legislation and aspects of published feline quality of life (QOL) assessment tools that fall under each need. Examples given are illustrative but not necessarily exhaustive

Five welfare needs	Additional context from QOL tools	References	Examples of purebred traits or management that increases the risk of compromised welfare and/or QOL	Examples of purebred traits or management where more research is required on potential welfare compromise
Need for a suitable environment	Clean litter box	Delgado & Reevy (2018)	–	
	Vertical territory	Delgado & Reevy (2018)		Stud cats; multi-cat breeding sites
	Scratching posts	Delgado & Reevy (2018)		Stud cats; multi-cat breeding sites
	Daily exercise	Delgado & Reevy (2018)	Indoor-only cats	Stud cats; breeding female cats
	Cared for when owner away	Delgado & Reevy (2018)	–	–
Need for a suitable diet	Clean food and water	Delgado & Reevy (2018)	–	–
	Appetite/difficulty eating	Niessen *et al.* (2010); Lynch *et al.* (2011); Freeman *et al.* (2012, 2016); Bijsmans *et al.* (2016); Tatlock *et al.* (2017)	Brachycephaly or other morphology impacting teeth; disease predispositions e.g. kidney disease	–
	Enjoying food	Freeman *et al.* (2012, 2016); Bijsmans *et al.* (2016)	Disease predispositions e.g. kidney disease	–
Need to be able to exhibit normal behaviour patterns.	Energy levels	Niessen *et al.* (2010); Lynch *et al.* (2011); Freeman *et al.* (2012, 2016); Tatlock *et al.* (2017)	Brachycephaly	–
	Play	Lynch *et al.* (2011); Freeman *et al.* (2012, 2016); Bijsmans *et al.* (2016); Noli *et al.* (2016); Tatlock *et al.* (2017); Noble *et al.* (2019)	Brachycephaly; painful conditions e.g. osteochondrodysplasia	Pain from shortened tail or dwarfism
	Sleep	Lynch *et al.* (2011); Freeman *et al.* (2012); Noli *et al.* (2016); Tatlock *et al.* (2017)	Brachycephaly or other respiratory issues; painful conditions e.g. osteochondrodysplasia	Pain from shortened tail or dwarfism
	Hiding	Freeman *et al.* (2012)	–	Painful conditions e.g. osteochondrodysplasia; decreased mobility
	Hunting	Noble *et al.* (2019)	Painful conditions e.g. from osteochondrodysplasia indoor only;	Hybrid breeds; Pain from shortened tail or dwarfism; brachycephalic cats
	Grooming	Lynch *et al.* (2011); Bijsmans *et al.* (2016); Tatlock *et al.* (2017)	Painful conditions e.g. osteochondrodysplasia	Shortened legs from dwarfism; divergent hair coats; pain from shortened tail or dwarfism
	Scratching claws	Bijsmans *et al.* (2016)	Painful conditions e.g. osteochondrodysplasia	Polydactyly; shortened legs from dwarfism; pain from shortened tail or dwarfism
	Kneading	Tatlock *et al.* (2017)	Painful conditions e.g. osteochondrodysplasia	Polydactyly; shortened legs from dwarfism; pain from shortened tail or dwarfism
Need to be housed with, or apart, from other animals.	Positive interactions with owner (affectionate, relaxed, friendly)	Lynch *et al.* (2011); Freeman *et al.* (2012, 2016); Bijsmans *et al.* (2016); Tatlock *et al.* (2017); Delgado & Reevy (2018)	Painful conditions e.g. osteochondrodysplasia	Behavioural traits; folded ears/no or shortened tail for social signalling; pain from shortened tail or dwarfism
	Negative interactions with owner (irritable)	Freeman *et al.* (2016)	Painful conditions e.g. osteochondrodysplasia; poorly socialised cats	Pain from shortened tail or dwarfism; behavioural traits; no or shortened tail for social signalling
	Interactivity with other pets	Freeman *et al.* (2012)	Painful conditions e.g. osteochondrodysplasia; poorly socialised cats	Behavioural traits; pain from shortened tail or dwarfism; folded ears/no or shortened tail for social signalling;
Need to be protected from pain, suffering, injury and disease.	Mobility—jumping, climbing	Lynch *et al.* (2011); Freeman *et al.* (2012, 2016); Bijsmans *et al.* (2016); Tatlock *et al.* (2017); Noble *et al.* (2019)	Painful conditions, e.g. osteochondrodysplasia; brachycephaly	Pain from shortened tail or dwarfism; shortened legs from dwarfism; lack of tail or shortened tail for balance
	Pain	Niessen *et al.* (2010); Lynch *et al.* (2011); Bijsmans *et al.* (2016); Tatlock *et al.* (2017); Noble *et al.* (2019)	Painful conditions e.g. osteochondrodysplasia	Pain from shortened tail or dwarfism
	Mood/happiness/personality changes	Niessen *et al.* (2010); Lynch *et al.* (2011); Freeman *et al.* (2012); Bijsmans *et al.* (2016); Noli *et al.* (2016); Tatlock *et al.* (2017); Noble *et al.* (2019)	Painful conditions e.g. osteochondrodysplasia; disease predisposition	Pain from shortened tail or dwarfism; behavioural traits
	Stress	Bijsmans *et al*. (2016)	Painful conditions e.g. osteochondrodysplasia; multi-cat breeding sites; indoor only cats; veterinary visits (for disease or genetic testing); pregnancy	Pain from shortened tail or dwarfism; behavioural traits
	Appearance (eyes, coat, weight)	Niessen *et al.* (2010); Freeman *et al.* (2016); Tatlock *et al.* (2017)	Disease predisposition; brachycephalism	Coat divergence
	Receives veterinary care	Freeman *et al.* (2012); Bijsmans *et al.* (2016); Noli *et al.* (2016); Delgado & Reevy (2018)	–	–
	Protected from hazards	Delgado & Reevy (2018)	–	–
	Health-related issues (e.g. medication, breathing, PUPD, diarrhoea)	Niessen *et al.* (2010); Lynch *et al.* (2011); Freeman *et al.* (2012); Bijsmans *et al.* (2016); Noli *et al.* (2016); Tatlock *et al.* (2017); Noble *et al.* (2019)	Painful conditions, e.g. osteochondrodysplasia; brachycephaly; predisposition to disease e.g. hypertrophic cardiomyopathy or kidney disease; pregnancy	Pain from shortened tail or dwarfism

Although the five welfare needs are a legal requirement in the UK, they are very broad as they apply to a wide range of species. Additionally, they reflect a minimum rather than a target standard of welfare. To better represent positive welfare experiences for cats, more specific feline welfare assessments were included. Several welfare assessment tools for owned cats have been published in the peer-reviewed literature, mostly titled ‘quality of life’ (QOL) assessments (for a review, see Doit *et al.* 2021). Although these tools were each designed for use on individual cats, the evaluations assessed within the tools can offer useful criteria on providing positive welfare experiences on innate health criteria, such as opportunities for play, grooming and scratching, and avoiding negative experiences. One specific tool lists cat care needs (Delgado & Reevy 2018), which is a useful lens when considering a suitable environment. Human perceptions and behaviours must also be considered for a fuller examination of purebred cat welfare, given they are central to cat husbandry and may influence ownership trends (Grigg & Kogan 2019; Plitman *et al.* 2019).

With this broad background, this review applied criteria from the current UK legislation bolstered by published cat QOL assessment tools to generate a framework that was then used to examine the welfare of cats, including physical and mental health, at a population level in the UK (Table 1). Using this framework, we specifically consider a range of welfare implications from being purebred in cats, including implicit risks from inherited disorders, morphology and behaviour, as well as breeding and husbandry conditions, with an evidence-based approach. This review is intended to highlight some prominent issues, but more exhaustive lists of breed-associated disorders can be found elsewhere (Gough *et al.* 2018; Nicholas *et al.* 2025).

### Extreme conformation

By definition, the morphology typical of each individual breed needs to differ sufficiently from the typical morphology of other individual purebred, crossbred or random-bred cats to ensure that each specific purebred breed is recognisable as a distinct entity, as previously defined in dogs (Worboys *et al.* 2018). To ensure this distinction, a range of conformational differences have been introduced by artificial selection of genetic variants that occurred spontaneously but had not become established in random-bred cats to create (i.e. invent) each new type of purebred cat (i.e. breed). These genetic variants were deliberately selected and preserved because they created phenotypes that were appealing to humans in some way. These novel phenotypes include, but are not limited to, variations in face shape and muzzle length, limb length, tail shape as well as in coat colour, texture and pattern. While there is clear evidence for health and welfare harms related to some of these novel phenotypes (Struck *et al.* 2020; Velie *et al.* 2023), some other novel conformational variants, such as coat pattern and colour, introduced to individual cat breeds have limited evidence supporting any negative welfare impacts. It should also be noted that some novel conformational variants which may negatively impact welfare are not limited to single individual breeds, such as the link between the dominant blue eyes/white fur combination and deafness (Bamber 1933; Abitbol *et al.* 2024).

**Figure 3.**
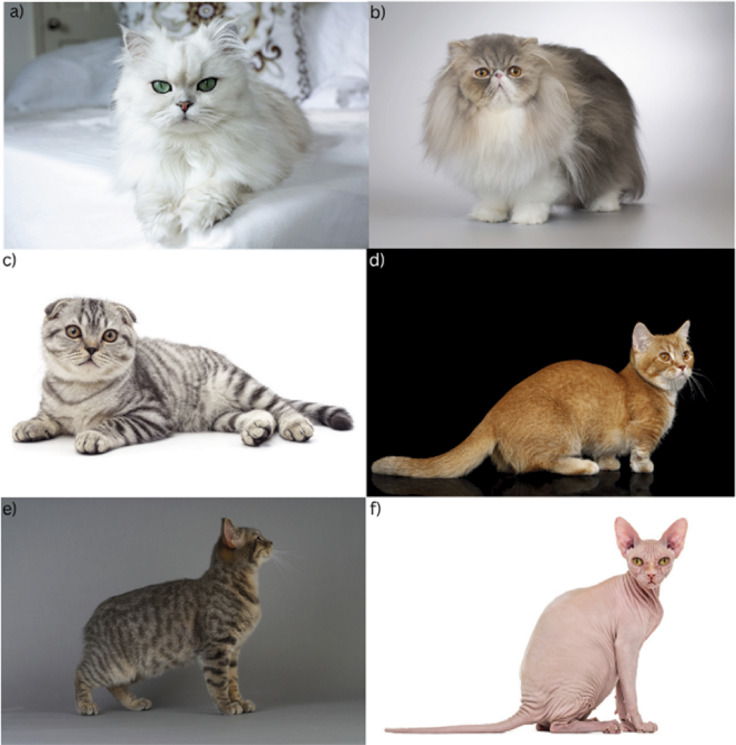
A selection of cats showing extreme conformation, including (a) Brachycephalic (‘Traditional’ Persian), (b) Hypertype brachycephalic (‘Peke-faced’ Persian), (c) Folded ears (Scottish Fold, also brachycephalic), (d) Dwarf cat (Munchkin), (e) Tailless cat (Manx cat), (f) Hairless cat (Sphynx). Credit: (a) iStock.com/Selcuk1; (b) iStock.com/Couperfield; (c) iStock.com/Voren1; (d) iStock.com/Seregraff; (e) iStock.com/Michael Viard; (f) iStock.com/GlobalP.

However, there is adequate evidence to show that several morphological differences that define certain current individual breeds are associated with substantial negative welfare impacts for cats and therefore can be classed as extreme conformation (Figure 1). Examples of extreme conformation that may impact welfare in cats include brachycephalism, folded ears, short limbs and lack of hair (Figure 3; Morel *et al.* 2024). Moving conformation further towards the extreme has become a norm in the show ring for dogs and cats, as judges often tend to prefer the more ‘spectacular’ hypertype conformation over the more innately healthy natural canine or feline conformation. In one survey, most dog show judges felt it was necessary to follow and promote adherence to current breed standards despite any intrinsic health issues associated with these interpretations, and despite most other stakeholders, including owners and breeders, largely disagreeing with this approach (Åsbjer *et al.* 2024). A small study on cat shows reported that judges of two brachycephalic cat breeds failed to note issues such as entropion that may impact welfare (Anagrius *et al.* 2021).

#### Brachycephaly

Brachycephaly (Figures 3[a], [b]) describes a distortion of skull morphology resulting in a flattened face with a shortened muzzle with other linked changes such as a shallower eye socket and altered cranial cavity (Künzel *et al.* 2003). Several cat breeds are considered brachycephalic (Table S1; Supplementary material), including British Shorthair, the most popular pure breed in the UK that makes up 8% of cats in the CATS Report 2024 (Cats Protection 2024) and 25% of all GCCF annual registrations (GCCF undated c). The distorted brachycephalic skull shape can cause painful conditions that may impact several of the welfare needs in Table 1, including ocular issues, such as entropion (Anagrius *et al.* 2021; Demir 2024) and conjunctivitis (Demir 2024), which may also result in corneal ulcers. Brachycephalic cats may have dental issues, including malocclusions (Mestrinho *et al.* 2018) and crowding (Mestrinho *et al.* 2018; Sieslack *et al.* 2021), which negatively affect their ability to eat (Gleason *et al.* 2023). Ability to eat is an important aspect of a suitable diet (Table 1). Hypertype brachycephalic cats include Persians with ‘Peke’ faces that are flatter than the traditional ‘doll-faced’, longer-nosed Persian cats (Figure 1; Morel *et al.* 2024). Peke faces (Figure 3[b]) are likely to exacerbate dental and respiratory issues (Farnworth *et al.* 2016). Doll-faced Persians are sometimes known as ‘traditional’ Persians to highlight changing show trends whereby breed standards have moved from promoting the longer noses in the original Persian appearance in the 1950s towards now promoting a more extreme flat-faced appearance (Morris 1999, cited in Schmidt *et al.* 2022).

As well as direct skeletal changes, brachycephalism may also cause respiratory problems stemming from relatively increased soft tissue in the shortened nose and also relative changes to the soft palate and larynx (Farnworth *et al.* 2016; Gleason *et al.* 2023) as well as stenotic nares (narrow nostrils; Razlighi *et al*. 2022. Consequently, brachycephalic cats may be more reluctant to exercise, recover more slowly from activity and have shorter activity periods than non-brachycephalic cats (Gleason *et al.* 2023). This is likely to impact on the ability to show some of the normal behaviours identified in the welfare framework (Table 1). There is currently limited evidence on other negative impacts of brachycephaly in cats, including impacts on behaviour, but brachycephalic dogs are shown to also have neuropathic pain and pain from reflux in the oesophagus (Mitze *et al.* 2022), as well as changes in their sense of smell (Polgar *et al.* 2016) and sight (McGreevy & Nicholas 1999), and reduced sleep efficiency (Roedler *et al.* 2013) so it is possible that similar effects could also occur in cats.

It is worth noting that some currently used methods of pain assessment based on facial expressions, including the feline grimace scale (FGS; Evangelista *et al.* 2019), did not include brachycephalic cats in their validation testing. As brachycephalic cats typically show greater pain-like features, especially Scottish Folds (Finka *et al.* 2019), these pain scales may be unsuitable for pain assessment in brachycephalic cats, and consequently brachycephalic cat breeds may be at more risk of pain going undetected.

#### Folded ears

Folded or curled ears define breeds such as the American Curl, Scottish Fold (Figure 3[c]) and Highlander breeds. In Scottish Fold cats, this deformity results from defective cartilage which is unable to hold the ear pinna upright (Allan 2000), with this defective cartilage also impacting bone development, termed osteochondrodysplasia (Chang *et al.* 2007). Scottish Fold osteochondrodysplasia may present as musculoskeletal abnormalities, such as exostoses (bone swellings) in the feet (Takanosu & Hattori 2020), and short, misshapen limbs and tails (Malik *et al.* 1999; Chang *et al.* 2007; Takanosu & Hattori 2020). As Scottish Fold osteochondrodysplasia is linked to the genetic variant which causes folded ears (Gandolfi *et al.* 2016), crossbred cats (e.g. cats with one Scottish Fold parent) with ear folds also have osteochondrodysplasia (Takanosu & Hattori 2020). Clinical signs of osteochondrodysplasia can begin as young as six months (Malik *et al.* 1999) and health status typically deteriorates with age (Malik *et al.* 1999; Allan 2000). Treatment for osteochondrodysplasia is never curative and may include surgical procedures that themselves can be painful and stressful, exacerbating the overall welfare impact. As with all painful conditions, osteochondrodysplasia is likely to inhibit natural behaviours, such as the ability to jump or climb as seen in the welfare framework (Table 1). Osteochondrodysplasia can also cause osteoarthritis, which is associated with significant chronic pain and restrictions in movement, including lameness, crawling gait and a reluctance to perform normal behaviours (Malik *et al.* 1999; Allan 2000). Osteoarthritis treatment and management is also not curative, and can include lifelong medication and environmental modification, such as steps or ramps, to enable cats to navigate their environment (Langley‐Hobbs 2023). Skeletal issues related to the ears in American Curls have not yet been reported (Takanosu & Hattori 2020) but further investigation is warranted.

Ears are important in feline communication: two cats meeting with both showing erect ears are significantly more likely to have positive interactions than those where either cat has non-erect ears (Deputte *et al.* 2021). It has not yet been reported whether cats with folded or curled ears, who are unable to extend their ears to erect have significant impacts on their ability to interact with other cats (Table 1). While stronger evidence on the social behaviour of cats with curled ears is required, the concerns around the wider welfare of cats with folded ears should be enough reason to recommend avoiding breeding for this conformation at all in cats.

#### Dwarf breeds

Munchkin cats (Figure 3[d]) and their descendant dwarf breeds typically have short limbs (Struck *et al.* 2020), although some offspring from breeding between two dwarf cats may display normal limb length (Lyons *et al.* 2019). Some genes that cause the shortening of limbs have been identified but are still under investigation (Lyons *et al.* 2019; Buckley *et al.* 2020). Although dwarf cats do not appear to share the high predisposition to intervertebral disc disease seen in dogs (Lyons *et al.* 2019), evidence is lacking as to whether the changes in skeletal morphology and resulting malalignment of limbs (Anderson *et al.* 2021) causes pain or predisposition to diseases such as the osteoarthritis seen in cats with dwarfism related to mucopolysaccharidosis (Allan 2000). To date, research appears limited to a single case study describing a Minuet with pectus excavatum (sunken breastbone), which compressed the thoracic contents and caused respiratory issues (Kihara *et al.* 2024). Research is also lacking on the extent to which short limbs *per se* impair natural behaviours, such as jumping, climbing and grooming. While population-level studies to provide evidence on welfare impacts in dwarf cats are warranted, it is worth noting that dwarf cat breeds, such as the Munchkin, are not currently recognised by GCCF or FIFe due to welfare concerns.

#### Congenitally tailless or short-tailed cats

Cats deliberately bred to have no tail, or a very short tail, such as the Manx (Figure 3[e]), suffer from wider vertebral deformities linked to this phenotype (Leipold *et al.* 1974; Deforest & Basrur 1979). Congenitally tailless or short-tailed cats carry a higher risk of spina bifida (Martin 1971), which shortens the nerves and spinal cord. As well as consequent issues with reduced and altered mobility, including paresis (muscular weakness) and/or a hopping gait (Leipold *et al.* 1974; Deforest & Basrur 1979), spina bifida in cats can also cause incontinence or other elimination problems (Leipold *et al.* 1974; Deforest & Basrur 1979). The mobility and elimination problems impact on cat welfare through the need for protection from pain and suffering and the need for a suitable diet (Table 1). Although much of the research on Manx cats dates back to the 1970s, and failed to investigate pain levels, it has been recommended that Manx cats should routinely undergo consideration of pain management (Lyons 2024). Further assessment of pain in breeds of cats with no or short tails is warranted.

Cats use their tails for balance (Walker *et al.* 1998). To our knowledge there has been no investigation as to whether having a short tail or no tail reduces a cat’s ability to balance and hence perform some of the natural behaviours in Table 1. Tails are also used in social interactions. The ‘tail up’ position for cats can be a friendly signal for an affiliative interaction, although it appears less important than the position of the ears as discussed above for cats with folded ears (Cafazzo & Natoli 2009). Cats usually approach humans with their tail up, which may reflect the way that kittens approach their mothers. The tail up is suspected to be an important part of the cat-human interaction (Deputte *et al.* 2021) and is theorised to be easily interpreted by humans as affiliative (Deputte *et al.* 2021). The impact of not having a full tail on the human-cat bond has been poorly investigated but could be relevant for the social needs in Table 1.

#### No hair coat or thin hair coat

Some cats have been deliberately selected to show genetic variants for hairlessness, such as Sphynxes (Figure 3[f]), or to have a very thin coat, such as Devon and Cornish Rexes. Cats with no or thin hair coats are reportedly prone to skin issues such as infection with the yeast Malassezia (Åhman & Bergström 2009), which can lead to dermatitis, causing itchy and inflamed skin and necessitating lifelong treatment with anti-fungal baths. Cats with no or thin hair coats are reported to be prone to skin damage from the sun or exposure to cold (e.g. People’s Dispensary for Sick Animals [PDSA] undated a) although robust evidence here is lacking. Given this possibility, owners may choose to restrict hairless cats indoors, reducing opportunities to perform normal behaviours and not providing a suitable environment if there is no appropriate enrichment as listed in the welfare framework in Table 1. Based on comprehensive reviews elsewhere regarding the welfare impacts and suitability of indoor-only lifestyles for cats (Foreman-Worsley & Farnworth 2019; Glanville *et al.* 2025), we note that enforced indoor-only living for cats is commonplace and the need for appropriate enrichment is not limited to any specific breed or to purebred cats more generally (Herron & Buffington 2010).

Although there appears to be no published information regarding the impact of no or a thin hair coat on grooming, Sphynxes are anecdotally prone to greasy exudate that accumulates on the skin and in the claws and interdigital web (Åhman & Bergström 2009). This may impact the need to be protected from pain, suffering, injury and disease as seen in the framework in Table 1. As a consequence to this exudate, the GCCF recommends regular bathing of Sphynxes (GCCF undated d), which itself can be a negative experience for cats (Rand *et al.* 2002). There are alternative and potentially less stressful options to bathing for cats, for example, using wipes instead of water. We also note that recommendations for grooming and bathing are not exclusive to hairless cats, and that cats with long hair coats also require frequent grooming to prevent potentially painful matting and reduce hairballs. Cat conformations that carry a requirement for human grooming to maintain skin and hair health could be considered an extreme conformation whereby if the human husbandry input is inadequate, then these animals will not have their welfare needs met.

#### Multi-extreme cats

Some cat breeds typically display more than one extreme conformation trait. For example, Scottish Folds typically have both brachycephaly and folded ears. Experimental breeds are often created by crossing cats from established breeds, resulting in planned selection for new cat types with multiple extreme conformations. For example, the Minuet is a Munchkin and Persian cross, resulting in a cat affected with both brachycephaly and dwarfism (TICA undated c). Recently, the XL Bully cat, a cross between Sphynx and Munchkin resulting in a hairless cat with skin folds and dwarfism, has attracted interest from animal welfare organisations as a breed of concern, although the Bambino, a similar cross, appears to have escaped such welfare attention (Ahmed 2024; The Cat Group 2024). Although the GCCF does not allow the registration of multi-extreme breeds, several are accepted by TICA (Table S1; Supplementary material).

#### Other conformational extremes in cats

In addition to the extreme conformations discussed above, humanity has selected for a wide range of other morphological changes in cats that do not commonly occur in natural cat populations, and which, as yet, have had very little research to establish their welfare impacts. These other phenotypes include polydactyly (extra digit[s] on the feet) and dolichocephaly, which refers to a longer face as seen in breeds such as Siamese. There is scant published research on polydactyly, but some lines of cats, including Maine Coons, are intentionally bred to maintain this trait (Hamelin *et al.* 2017). The extra digit causes changes in the carpal and tarsal bones in the foot, although the authors of that study concluded, based on their available evidence, that “*polydactyly had no impact on cat welfare*” (Hamelin *et al.* 2017). However, further research is warranted to better assess the impact of polydactyly on welfare, including analyses with larger samples with more lines of cats, more breeds, older study populations, and recognition of the diverse conformation polydactyly may take. Likewise, there is no evidence published to date on welfare impacts of dolichocephaly on health, behaviour or social signalling, including those with the extreme features of hypertypes, such as large ears (Morel *et al.* 2024), although this does not mean impacts do not exist.

### Breed predispositions to disease

Protection from disease is explicit in the five welfare needs (Table 1). Several published QOL assessment tools also include a strong health-related component and focus on specific indicators and consequences of disease from clinical signs, such as breathing difficulties, increased urination and diarrhoea (see Table 1). In addition to welfare impacts from primary clinical signs, diseases may impact upon mood or appetite and can require medications and veterinary visits for tests and check-ups which add additional potential sources of stress in the welfare framework (Table 1). Predispositions to disease have been reported in several cat breeds, with these diseases often remaining stubbornly prevalent within breed-types over time due to the small genetic pools available for some breeds. Illustrative examples to demonstrate disease impact on welfare are discussed below; for a more complete review see Gough *et al.* (2018) and Lyons (2024). As disease predisposition is such a broad topic with many breeds to be considered, numerous areas are lacking research and much of the published research is not recent. We also note that predisposition to disease reflects an increased risk of disease but does not mean that every cat within a certain breed or phenotype will develop that disease and, in some cases, environmental and other factors may also increase risk.

#### Painful conditions

The need to be protected from pain is integral to the legally defined feline welfare needs. Indicators of pain in cats utilised in QOL assessment tools include yowling, changes in mobility and reluctance to jump (Table 1). Although acute and chronic pain can be managed to some degree with appropriate analgesia, pain itself can be difficult to detect in cats, and not all medications are highly effective with some potentially carrying serious side-effects (Taylor & Robertson 2004). Chronic pain and its impact on mobility may also require management via environmental modifications, such as steps or changes to litter tray accessibility.

Painful conditions associated with purebred cat breeds include diseases of the urinary system such as urolithiasis that is predisposed in Bengals, Birmans, Egyptian Maus and Exotic Shorthairs, among others (Albasan *et al.* 2012). Skin issues, such as an increased incidence of atopic dermatitis in Abyssinians (Ravens *et al.* 2014), may lead to painful lesions. Painful oral conditions, such as tooth resorptive lesions (Vapalahti *et al.* 2024) and periodontal disease (Lund 2012; O’Neill *et al.* 2023a) are more common in various breeds, and may reduce appetite or the ability to eat, impacting on the welfare need for a suitable diet (Table 1).

Feline hip dysplasia is a rarer painful inherited condition defined by malformation of the coxofermoral (hip) joint (Keller *et al.* 1999). Hip dysplasia in cats is poorly recognised and understood but appears to be more common in Maine Coons, Himalayans and Persians (Keller *et al.* 1999; Loder & Todhunter 2018). As well as causing primary pain, hip dysplasia can lead to further pain from secondary osteoarthritis (Allan 2000). Hypertype Maine Coons may be larger-bodied than typical Maine Coons (Morel *et al.* 2024) with the potential to exacerbate the clinical signs of both hip dysplasia and osteoarthritis.

#### Other breed predispositions

Hypertrophic cardiomyopathy (HCM) is the most commonly reported heart disease in cats and is more prevalent in Maine Coons, Persians, British Shorthairs and Ragdolls (Granström *et al.* 2011; Trehiou‐Sechi *et al.* 2012; Casamian‐Sorrosal *et al.* 2014). HCM can occur from six months of age (Kittleson & Côté 2021) but may have a later onset of 6–7 years (Kittleson *et al.* 1999). In HCM, the heart muscles become thickened, reducing their efficiency. Although many cats with HCM can live an apparently normal life (Maron 2018), clinically affected cats can show serious clinical signs including difficulty breathing, anorexia, hind leg paralysis and sudden death. A QOL assessment tool exists specifically for cats with cardiac disease (Table 1; Freeman *et al.* 2012) which includes questions on breathing, collapse, increased drinking/urination, appetite changes, medications and veterinary visits, highlighting the wide spectrum of welfare impacts that cardiac disease may have on QOL. Variants responsible for HCM have been identified (Meurs *et al.* 2009), with genetic testing for HCM as a familial trait available for certain breeds, such as Maine Coons and Ragdolls (Langford Vets undated). It is advised that breeding cats are screened using echocardiography (Häggström *et al.* 2015), meaning potential stressful veterinary visits impacting on the welfare need to be protected from pain, suffering, injury and disease (Table 1).

Polycystic kidney disease (PKD) is a familial disorder identified in Persians (Biller *et al.* 1996) and in breeds derived from Persians, including Exotic Shorthairs and Himalayans. A genetic test is available for the causative gene (Langford Vets undated) which has been identified (Lyons *et al.* 2004). PKD is present at birth and progresses with age, with renal function deterioration generally resulting in clinical signs that typically appear at around seven years of age, although they can begin between three and ten years (Schirrer *et al.* 2021). Clinical signs include increase in thirst and urination, weight loss, vomiting/nausea and death which can impact on several welfare needs (Table 1). Treatment is palliative and might include fluid therapy, anti-nausea medications and dietary changes. In 2001, the prevalence of PKD in Persians in the UK was 49.2% (Cannon *et al.* 2001). To our knowledge, a more recent published estimate is not available and although the GCCF reports a decrease in PKD prevalence since the onset of the genetic test, it is not clear where those data were sourced (GCCF undated e). Widespread genetic testing is reported to have contributed to reduced prevalence of PKD in other countries such as Japan, although at this time these data are not yet peer-reviewed (Ukawa *et al.* 2024). The causative PKD1 gene was not found in a more recent sample of 118 Persian cats from around the world (Anderson *et al.* 2022). An updated investigation into the epidemiology of PKD in Persians and other breeds in the UK would be valuable.

Genetic testing of a large global sample of cats has indicated that several other genetic variants associated with disease have also decreased (Anderson *et al.* 2022). Although the link between these decreases and the onset of genetic testing is not proven, it seems a likely explanation, and it has been postulated that many specific disorders could be reduced or even eradicated from pedigree lines if genetic testing were more widespread or even mandatory (Lyons 2024). To our knowledge there is no current evidence regarding the levels of genetic testing of cats in the UK, although it is mandatory for some breeds registering with the GCCF.

Diabetes mellitus is a chronic endocrine disease of relative insulin insufficiency that generally requires daily insulin injections for clinical management, although there is now an oral solution available for newly diagnosed cats. Diabetes predisposition is reported in Burmese and Russian Blue cats, among others (Öhlund *et al.* 2015), and evidence supports a genetic predisposition (O’Neill *et al.* 2016). However certain purebreds, including Bengals, Persians, Ragdolls and British Shorthairs show a lower risk of developing diabetes mellitus compared with random-bred cats (Öhlund *et al.* 2015). Random-bred related factors, such as age, obesity, being inactive and treatment with corticosteroids can also put cats at higher risk of developing diabetes (McCann *et al.* 2007; Öhland *et al.* 2015). There is a QOL tool specific to cats with diabetes mellitus, with questions regarding increased appetite, being unwell in general and pain from insulin injections (Table 1; Niessen *et al.* 2010).

Feline infectious peritonitis (FIP) is a viral disease, with young and male cats overrepresented (Worthing *et al.* 2012), alongside several breeds, including Abyssinians, Bengals, Birmans, Himalayans, Ragdolls and Rexes compared to the random-bred cat population (Pesteanu-Somogyi *et al.* 2006; Worthing *et al.* 2012). Clinical signs are varied and can include abdominal effusion, fever, anorexia and neurological signs (Tasker *et al.* 2023). FIP carries a very poor prognosis, with one study reporting median survival times of 21–38 days after presentation (Tsai *et al.* 2011). Since 2021, antiviral medication has been legally available in the UK to treat FIP (Taylor *et al.* 2024). This is typically a 12-week course of tablets (Coggins *et al.* 2023). During treatment cats may suffer pain, need fluid drained from the lungs and/or experience side-effects of medication, such as nausea (Taylor *et al.* 2024).

### Behavioural traits

Individual cat breeds may also be associated with greater display of certain behavioural traits, such as aggression towards other cats or humans, many of which may be linked to extreme conformation (Wilhelmy *et al.* 2016; Salonen *et al.* 2019). These traits may require specific management to provide cats with a suitable environment, or the need to be housed with or apart from other animals (Table 1). Feline behavioural science has been subject to much less research over past decades than issues related to physical health, and many published studies show heavy bias by typically being based on owner-reported information (Marsh & Hanlon 2007). For example, owners who chose their cat breed based on anecdotal breed-typical traits, such as Ragdolls being affectionate and laid-back (GCCF undated f), may then be more likely to report these traits in their cat based on their prior beliefs. Feline behaviour is highly complex, situational and multifactorial, and is heavily linked to socialisation, early life experiences, environment and other factors (e.g. McCune 1995; Casey & Bradshaw 2008; Foreman-Worsley & Farnworth 2019; Eagan *et al.* 2021; Campbell *et al.* 2024), of which breed is just one. Some unwanted behaviours may be also linked to other underlying welfare issues that might be associated with purebred cats, such as pain (Mills *et al.* 2020).

#### Cat-directed aggression

Appropriate application of the legal welfare need to be housed with or apart from other animals (Table 1) may vary between individual cats. Some breeds have been associated with higher levels of cat-directed aggression, such as Abyssinians, Tonkinese, Oriental Shorthairs (Wilhelmy *et al.* 2016) and Turkish Vans (Salonen *et al.* 2019). If true, individual cats of these breeds may be better suited to single-cat households. Other breeds, such as Persians, show reportedly lower frequency and severity of cat-directed aggression (Salonen *et al.* 2019) and therefore may more readily cohabit with conspecifics. However, as noted above, these studies are based on owner reports and are insufficient for making recommendations based on breed. Additionally, individual personality and socialisation should always be considered when assessing suitability for multi-cat households. Bengals, a hybrid breed, have been reported to not have an increased incidence of cat-directed aggression (Wilhelmy *et al.* 2016; Salonen *et al.* 2019). However, hybrid cats generally are reported anecdotally as being more likely to have negative interactions with other cats, especially earlier hybrid generations which are closer to original wild cats (AAFP 2017). More robust behaviour studies incorporating more hybrid breeds would be valuable in decision-making around multi-cat households. There appears to be no evidence regarding whether any specific cat breeds are more or less amenable to living with other companion animals such as dogs.

#### Relationship with humans

Some individual cat breeds are also reported to show differing levels of human-directed aggression, although the literature lacks consistency regarding many of these associations. Birmans, Maine Coons and Turkish Vans have been reported to show higher levels of aggression towards familiar humans, while British Shorthairs, Maine Coons and Persians reportedly exhibited lower aggression (Wilhelmy *et al.* 2016; Salonen *et al.* 2019). However, more generally, ‘mixed-breed’ (assumptively random-bred) cats were more likely to act aggressively towards humans than purebred cats in one study (Ramos & Mills 2009). All three of these studies are based on owner-reported behaviour. Desirable behaviour towards humans is important for cat welfare as a strong bond between owners and companion animals has been found to improve many indicators of good pet care, including a higher likelihood of seeking veterinary care (Lue *et al.* 2008). Positive interactions with an owner (e.g. affectionate, relaxed behaviours) are often used as QOL indicators in assessment tools (Table 1).

#### Environmental needs

Environmental needs may vary predictably between breeds. Bengals, Birmans and Persians have been associated with higher incidences of inappropriate elimination, which might include urinating outside the litter tray and marking behaviour (Amat *et al.* 2009; Wassink-van der Schot *et al.* 2016; Wilhelmy *et al.* 2016). The reasons for this association are unclear; underlying causes for not using the litter tray include litter type and location (Horwitz 1997; Herron 2010), multi-cat households and lack of outdoor access (Barcelos *et al.* 2024), as well as health issues such as cystitis which can be related to stress in cats (Buffington 2002). Urine marking, (also known as spraying), is a physiologically normal but unwanted behaviour linked with being entire, particularly in males (Pryor *et al.* 2001), as well as the age and personality of a cat (Barcelos *et al.* 2024). Cats exhibiting inappropriate elimination may need environmental modification to address the underlying issue, such as alternative litter tray provisions and management of agonistic social interactions (Horwitz 1997).

Individual cats of other breeds may also require specific environmental consideration to fulfil needs that are potentially more complex. Hybrid cats (Figure 1, Table S2; Supplementary material), are likely to retain strong natural hunting instincts and temperaments that require more mental and physical enrichment to fulfil. Although this has not been studied for all hybrids, Bengals have been reported to exhibit higher levels of hunting behaviour and are highly active (Wilhelmy *et al.* 2016; Salonen *et al.* 2019). Many hybrids appear to have specific needs that often make them unsuitable for typical home environments, and therefore many UK cat welfare charities do not advocate their breeding (Cats Protection undated; International Cat Care undated). Despite these welfare considerations, Bengals are reported to be increasing in popularity in the UK (Cats Protection 2024), although the numbers of other hybrids kept as companion animals in the UK is unknown. More work is therefore needed to understand the behavioural traits and environmental needs of hybrids and whether these can be adequately met in the home as required by the framework in Table 1.

### Breeding practices

To our knowledge, there have yet to be any UK studies published investigating the welfare impacts on purebred cats in terms of breeding management, although some information is available from a Swedish study (Ström Holst & Frössling 2009). Scotland has introduced The Animal Welfare (Licensing of Activities Involving Animals) (Scotland) Regulations 2021 that requires a licence for anyone breeding three or more cat litters over 12 months. The rest of the UK currently has no legislation or distinction between commercial and non-commercial breeders. The European Parliament and the Council on the welfare of dogs and cats have also recently announced plans for mandatory registration for cat breeding establishments in the EU that keep three or more queens and producing in total three or more litters per establishment and calendar year (European Union 2025). Although accidental breeding may occur in purebred cats, this section focuses upon welfare in the context of intentional breeding, including pregnancy and kitten welfare.

#### Housing

The living conditions for breeding cats in the UK have not been reported. However, these conditions are important to consider carefully, given their ability to impact upon ability to fulfil the legal needs for a suitable environment, the need to be housed with, or apart from, other animals, and the need to be free from disease as listed in the framework (Table 1). There are no reliable data regarding how many cats reside in breeding catteries in the UK, nor how many cats are typically kept at any one site, although evidence from international studies suggests multi-cat breeding sites are common. In Sweden, the mean number of cats per breeding site was reported at six (Ström Holst & Frössling 2009), while in Germany the reported median was 12 cats, with a range of five to 29 (Klein-Richers *et al.* 2020). Many of these breeding cats cohabit, with 74% of Swedish breeding catteries housing their cats together (Ström Holst & Frössling 2009). Enforced living in a multi-cat environment is recognised as a potential source of stress for cats in the framework in Table 1, although a recent review found that reported links between stress and multi-cat households vary considerably across studies (Finka & Foreman-Worsley 2022).

Goericke-Pesch and Packeiser (2022) reported that breeding queens are often kept in the house as pets, with breeding tom cats kept individually in a room or in a ‘garden house’. Limited space and poor enrichment may restrict the natural behaviours listed in Table 1. Multi-cat environments are also linked with higher risks of infectious diseases, for example, Feline Calicivirus and Feline Herpesvirus appear to be more common in multi-cat than single-cat households (Binns & Dawson 1995) and in Sweden, 33% of breeders reported having a cat with conjunctivitis within the past year (Ström Holst & Frössling 2009). Breeders may find it difficult to balance between hygiene to reduce the risk of infectious disease spread and complying with any licencing laws while still providing for the environmental needs of cats, such as consistent and predictable human-cat interactions (see Ellis *et al.* 2013).

#### Pregnancy

Pregnancy itself can be a welfare concern. Dystocia, defined as an obstructed or difficult birth, appears to be significantly more common in some cat breeds, including Abyssinians and British Short Hairs, based on insurance data (Ström Holst *et al.* 2017). Dystocia is likely to cause pain and distress, and can lead to the death of the queen, kittens or both (Ström Holst *et al.* 2017; Černá *et al.* 2024), impacting on the need to be protected from suffering, pain injury and disease in the welfare framework (Table 1). Dystocia may require a Caesarean section with associated risks of surgery and anaesthesia (Ström Holst *et al.* 2017). In a 1994 study from one Swedish veterinary hospital, surgical intervention was undertaken in 79.4% of feline dystocia cases (Ekstrand & Linde‐Forsberg 1994). There is also evidence that pelvic measurements are significantly smaller in brachycephalic cats in all dimensions, including a smaller pelvic canal (Monteiro *et al.* 2013), although the impact of this on the incidence of dystocia is not clear. Gunn-Moore and Thrusfield (1995) reported a higher incidence of dystocia in brachycephalic cats, while Ström Holst *et al.* (2017) found the incidence to be higher in some brachycephalic breeds, such as British Shorthairs, but lower in others, including Persians. It should also be noted that breeding cats must be left entire which can increase the risk of health issues including mammary cancer (Overley *et al.* 2005) or pyometra (Hagman *et al.* 2014).

#### Socialisation

Effective socialisation of kittens in a breeding facility may be difficult, and in some cases neglected, although the evidence base on socialisation in breeding facilities is limited. The socialisation period of kittens is thought to be between two and seven weeks (Karsh & Turner 1998) with kittens requiring exposure to experiences, including sights, sounds and interactions with other species, such as cats, dogs and humans, in their early life to ensure they develop the required skills to cope in domestic settings as they age (McCune 1995; Casey & Bradshaw 2008; Campbell *et al.* 2024). Where this socialisation does not take place adequately or at all, cats may struggle to cope later with household environments, impacting on the ability to provide a suitable environment and need to be housed with or apart from other animals (Table 1).

#### Hybrid cats

Special consideration must be given to the deliberate breeding to produce hybrid cats (Table S2; Supplementary material). Breeding domestic cats with wild cats may risk severe injury or death to the domestic cat. Wild cats kept for breeding are often housed in unsuitable domesticated situations for extended periods and may have been removed illegally from the wild (AAFP 2017). First generation crossings can produce infertile males (Gershony *et al.* 2014) that are unsuitable for breeding and likely unsuitable as companion animals. Although the welfare outcomes for these males are not reported, relinquishment or euthanasia seems likely. In some countries, concerns regarding hybrid cats have led to bans on their importation, for example, New Zealand only allows importation of Bengals of at least F5 or later, and no other hybrids are allowed to be imported (New Zealand Government 2024). Although not banned in the UK, any cat with a wild parent requires a licencing permit under the Dangerous Wild Animals Act 1976 (UK Parliament 2022). There is variation across UK cat registration bodies in the acceptability of hybrid cats. The GCCF does not allow registration of early generation (F1–F3) Bengals (GCCF 2017). The Toyger, which is derived from Bengals, is recognised as a preliminary breed (in the early stages of acceptance, see Table S1; Supplementary material). FIFe only allows registration of Bengals but do not allow breeding from generations F1–F4 (FIFe 2025), while TICA recognises four hybrids (Bengal, Chausie, Savannah and Toyger, see Table S2; Supplementary material), information on accepted generations could not be readily found. We note that the Highlander cat, recognised only by TICA, has been reported by some sources (e.g. Rare and Exotic Feline Registry undated) to be derived from hybrid cats although TICA describes them as being derived “*from the domestic gene pool*” (TICA undated d). Other hybrids (Table S2; Supplementary material) are not recognised by any UK registration body, nor are hybrids recommended as pets by many animal welfare organisations (e.g. Cats Protection undated; PDSA undated b).

### Human behaviour

The many potential negative impacts of our human behaviours on the welfare of purebred cats have not been well researched. The human behaviours considered here include aspects of husbandry, veterinary care and diet. Consideration is also given to motivations behind purebred cat acquisition and acceptance of extreme features despite their potential negative welfare impacts on cats. Several specific areas are highlighted which would benefit from more research to facilitate interventions that encourage human behaviour change and improve welfare of purebred cats.

#### Husbandry and management

It is likely that breed impacts on indoor-outdoor status, with one global study reporting ‘pedigrees’ (assumptively purebreds under the definition in Figure 1) were significantly more likely than ‘non-pedigrees’ to be enforced indoor-only, making up 16% of the indoor only population compared to 7.9% of the population allowed outside (Foreman-Worsley *et al.* 2021). Crossbred cats were not reported but are likely to fall under the umbrella of ‘pedigree’ since they have purebred ancestry. Reasons for keeping their cats inside cited by owners of purebred cats included concerns about theft, stipulation from the breeder and impressions that their cat was not able to keep itself safe outdoors (Foreman-Worsley *et al.* 2021). Although an indoor life provides some protection against outdoor hazards such as cat fights and road traffic collisions (Tan *et al.* 2020), outdoor access allows cats more space and access to resources that enable performance of natural behaviours, including hunting and ranging (Bradshaw 2018). Outdoor access also provides opportunities for exercise, which may explain higher levels of obesity seen in indoor-only cats than those with outdoor access (Buffington 2002; Rowe *et al.* 2015; Wall *et al.* 2019). However, humans are more likely to provide indoor cats with better indoor enrichment such as toys and scratching posts (Lawson *et al.* 2020; Machado *et al.* 2020; Tan *et al.* 2020) that may mitigate some of the welfare compromise. Across the spectrum of purebred cats, it is unclear whether some breeds are more affected by human decisions regarding cat lifestyle than others, although it may be rationalised that high-energy breeds and hybrid cats would benefit more from increased opportunities to be active and fulfil their natural instincts, such as through play. In the absence of literature in this area, it is important that owners understand the individual needs of their cat regardless of breed and are able to provide for them without unacceptably compromising welfare.

#### Travel

There is very scant evidence published on the frequency of travel and the distances undertaken for breeding purebred cats or displaying them at shows. In Sweden, most breeders (87%) reported showing a cat at least once in the previous year, with this extending up to 26 shows annually for some breeders, although it is not clear how many shows this is for each cat (Ström Holst & Frössling 2009). Travel itself is a potential source of major stress to cats, although most research to date has focused on travel specifically for veterinary visits (e.g. Mariti *et al.* 2017; Tateo *et al.* 2021). Mixing with other cats when breeding or at shows may also increase the spread of disease; 10.4% of Swedish breeders reported a cat having conjunctivitis after travelling to a show and 14.6% reported signs of upper respiratory tract disease (Ström Holst & Frössling 2009). Additional issues for show cats may include the stress of encountering unfamiliar cats, being confined to a small crate for extended periods, limited hiding places, exposure to unfamiliar humans and handling, noise and unfamiliar odours (Stone 2019). To our knowledge, only one study has investigated stress at cat shows, and although the authors of that study reported only low levels of behaviours that indicated stress, they noted that this was only a pilot study (Cannas *et al.* 2023). More robust work is needed to assess how showing of purebred cats impacts on the welfare framework in Table 1.

#### Veterinary care

Veterinary care is an important aspect of maintaining the welfare needs in the framework in Table 1. Given the increased risk of health and pain issues discussed above for some specific cat breeds, some purebred cats may require increased levels of veterinary care compared to random-bred cats. For this, owners must firstly be sufficiently aware of the status of their cat’s health. Unfortunately, normalisation of suffering related to breed-typical extreme conformation has been reported as widespread among owners of purebred dogs, with owners of brachycephalic dogs reported to often be unaware of the severity of their dog’s respiratory clinical signs, instead believing them to be ‘normal for breed’ (Packer *et al.* 2012). It is likely that a similar cognitive dissonance and bias could prevail for owners of purebred cats. Once an awareness of disease has been reached, owners must then also be willing and able to obtain veterinary care and to undertake to administer any necessary treatments and make suggested changes to their cat’s environment.

There is some evidence suggesting that the owners of purebred cats provide more veterinary care than owners of random-bred cats. For example, in Denmark (Sandøe *et al.* 2017b) and Chile (Salgado-Caxito *et al.* 2023), levels of vaccination were reported to be higher in purebred cats than in random-bred cats. Although there appears to be limited peer-reviewed studies in the UK, data collected by Cats Protection in 2024 as part of a survey of over 6,000 cat owners showed that purebred or pedigree cats had slightly higher levels of vaccination (80 vs 74%) than random-bred cats (unpublished data). There was no difference reported in the proportion of cats who were registered for veterinary care (both 92%), but more pedigree and purebred owners reported their cat having a regular check-up (70 vs 60%) and having their cat insured (62 vs 45%) than owners of random-bred cats (unpublished data). Whether these differences are statistically significant or impacted by other factors (for example, indoor-outdoor status) is unknown but it may also result from some purebred cats requiring increased healthcare. Insurance prices are generally higher for purebred cats, and this is often related to an increased need for healthcare (McEntee 2021; Go Compare 2024). More information on levels of veterinary care both needed by and provided to purebred cats is needed.

#### Diet

To achieve the welfare need for a suitable diet, cats are required to be provided with adequate food but also need to be able to eat normally and maintain a good appetite (Table 1). While there is limited published information regarding whether different breeds of cats have different nutritional needs, at least one cat food company markets breed-specific foods (Royal Canin undated). Although these breed-specific foods may, for example, claim to cater to the shape of a cat’s jaw, their hair coat type, or known health associations, such as cardiac issues, there is limited evidence on the specific benefits of these products. Additionally, it is unclear the extent to which catering to specific conformations perpetuates the normalisation of extreme traits.

There may be an association between purebred cats in general and the feeding of raw food diets (O’Halloran *et al.* 2021). For example, an outbreak of *Mycobacterium bovis* was seen in 47 cats that had been fed a commercial raw diet, where 76% of the cats were purebred or crossbred cats of various breeds (O’Halloran *et al.* 2021). Sarcocystis and toxoplasmosis have also been identified in raw food (van Bree *et al.* 2018). Other issues with raw diets are a potential lack or imbalance of essential nutrients, such as taurine, which are typically supplemented in complete commercial diets. With little evidence on raw food diets, recommendations are difficult to make, although it seems reasonable to assume that commercial raw diets are more likely to have complete nutrients and higher health and safety standards than home-made diets.

#### Motivations behind purebred cat acquisition

A review of human behaviour would not be complete without mention of the motivations for owning a purebred cat and insight into the acceptance and desirability of extreme features of breeds like brachycephaly despite their negative impact on welfare. Personal justification and rationalisation for breed choice will likely vary across breeds as well as between individual humans, as seen with dogs (Sandøe *et al.* 2017a). However, there is some evidence that appears to show similar drivers of human choice for cat breeds as for dog breeds, with key human focus on perceptions around appearance (Plitman *et al.* 2019) and personality (Berteselli *et al.* 2023), while good health is deemed of less importance (Plitman *et al.* 2019). Human social status may also be an influence for choice of cat breeds, as seen for dog breeds; owners of brachycephalic dogs are significantly younger, in the age groups 18–24 and 25–34 (Packer *et al.* 2017). For cats, reports similarly show that humans in the age group 18–34 are more likely to own a purebred or pedigree cat of any breed (Cats Protection 2024).

Packer *et al.* (2017) hypothesised that a dependence on social media in younger age groups was a potential driver to own brachycephalic dogs. The same has been proposed anecdotally for an increase in purebred and pedigree cats by cat welfare organisations (Cats Protection 2023), as well as the use of particular cat breeds in advertising (International Cat Care 2023). Social media increases the visibility of purebred cats, it may also inspire the purchasing of a specific breed: 29% of pedigree cat owners in one survey chose the cat for ‘likes’ on social media, with 5% of non-purebred cat owners saying the same (Cats Protection 2023). Social media, along with other information dissemination networks, can spread misinformation on cat breeds, including perceived personality traits and also offer routes to buying and selling cats. With the internet and its trends constantly changing, research is lagging behind in multiple areas, including new breeds and acquisition trends and motivations.

Preference for individual breeds may also have a cultural influence. For example, respondents who lived in Asia completing a survey on breed preference were more likely to prefer brachycephalic and dolichocephalic cats than mesocephalic, compared with respondents in the rest of the world (Farnworth *et al.* 2018). Similar to owners of brachycephalic dogs, owners of brachycephalic cats appear to be highly unaware of prevailing health issues in these cats (Berteselli *et al.* 2023), and so increased public education around this may be a route to behaviour change. However, a survey found that almost one in seven owners of extreme brachycephalic dogs reported that nothing would dissuade them from owning a flat-faced dog (Packer *et al.* 2024). In these cases, education alone would be unlikely to be a sufficient intervention and instead the powers of legislation or removal of the ‘social licence to own’ may need to be invoked to protect the welfare of these cats from well-meaning but misguided humans (Beban *et al.* 2024; LAGECDogs 2024). Whether owners of brachycephalic cats would report similar views is unknown.

### Animal welfare implications

This Horizon Topic paper has reviewed the available evidence on a wide range of elements related to the welfare of purebred cats relative to random-bred cats, with consideration also given to crossbred cats. A framework was designed based on the current legislation and supported by QOL tools. Several factors are identified that raise serious health and welfare concerns for cats that humans deliberately produce to meet the typical phenotype and behaviours of these purebreds. Many of these welfare concerns also contravene the legislation set out by the Animal Welfare Acts and impede the QOL of these cats (Table 1). Key areas of concern include extreme conformation; physical traits that impede the ability of cats to eat, drink, breathe properly or behave normally. From a welfare and also legislative, moral and ethical perspectives, it is clear that it should no longer be considered acceptable to deliberately produce cats with extreme traits that negatively affect welfare; for example, there should be an immediate and complete cessation of breeding of cats with folded ears. For traits where the evidence on the welfare impact is less robust, making specific recommendations for improving the welfare of affected purebred cats in the UK is likely to be more nuanced and, as we have identified, in many areas requires further research and evidence. Overall, cat welfare could benefit from borrowing the global public advice given for dogs to ‘Stop and think before acquiring an animal with extreme conformation’ (ICECDogs 2024). It should be noted that this welfare advice applies to the conformation regardless of the pure breed status and should also apply to crossbred cats with these same extreme phenotypic traits. Indeed, some crossbreds have multiple genetic variants that may mean their welfare is even worse than for some purebreds.

The current review also identifies that a greater understanding of the management of purebred cats in the UK is needed, including breeding practices, husbandry, travel and provision of veterinary care. Much of these practices currently seem to be largely unmonitored and unregulated, and there could be major welfare issues that are as yet unrecognised. It can then be identified where these aspects may fall short or indeed, as with veterinary care, whether purebred cats may sometimes even be at an advantage over random-bred cats. In addition, the cat QOL assessment tools referenced in this Horizon Topic paper (Table 1) could be explored for use on individual cats to assess the impact that the genetic and morphological features discussed have on welfare. To our knowledge none have yet been explored for use in this way.

Human behaviours were a key area which the current review identified with substantial scope for improvement to protect purebred cat welfare. The limited evidence on what, why and how these behaviours arise and are actioned was flagged as an information gap that urgently needs to be filled. Within these key data gaps is a need to understand the human motivation for desiring or acquiring cats with extreme traits at all. Given the known health concerns of many purebred cats, it would be beneficial to understand whether owners are aware of these welfare issues or not prior to acquisition. Only then can humanity truly act to change these behaviours to more welfare-friendly approaches based on a solid evidence base. One aspect that this review has not identified is which stakeholders hold the greatest power to create this change. For dogs, this power was historically placed with the Kennel Club who dictate the breed standards to which breeders must adhere. Veterinary surgeons and animal welfare organisations have also held some power to highlight their concerns (e.g. BVA 2018). However, more recently, increasing levels of acquisition of dogs with extreme conformation, such as French Bulldogs and designer breeds such as Cockapoos that are not promoted by the Kennel Club or veterinary bodies, suggests that the historic power of large bodies such as these has either dissipated or was always illusory in the first place (O’Neill *et al.* 2023b). Instead, in parallel to the situation for dogs, it could be argued that the power for positive welfare change now lies mainly with the wider public, who ultimately make the important decisions on what type of cat they acquire and through which route, and whether they breed from their cat and take part in cat shows. And with this great power comes great responsibility to prevent these breeding, acquisition and management decisions leading to unnecessary suffering for these cats. Consequently, interventions such as educating the general public on key issues of cat welfare (such as those covered by this paper) will be critical in protecting the welfare of purebred cats.

Over recent years, the rapid escalation of human desire to own dog breeds with brachycephaly has meant that interventions to protect dogs from human behaviours have focused heavily on brachycephalic breeds (Packer & O’Neill 2021). These efforts have included health testing and the puppy contract aimed at reducing the frequency and severity of health issues (AWF & RSPCA 2018) and legal interventions such as those seen in The Netherlands to prevent breeding of dogs with muzzles that are too short (Van Hagen 2019). In the UK, the Brachycephalic Working Group (BWG) as a collaborative between academia, the government, breed clubs and the Kennel Club, the veterinary profession and animal charities aims to work towards improved health in brachycephalic dog breeds (BWG 2024). The UK Legal Advisory Group on Extreme Conformation in Dogs (LAGECDogs) comprises legal and welfare experts with a vision for a world where every domestic dog is born free from extremes of conformation that harm their health and welfare (LAGECDogs 2024). Since the international movement of dogs means that the welfare issues and zeitgeist human thinking related to conformation of dogs is now a global phenomenon, the International Collaborative on Extreme Conformation in Dogs (ICECDogs) has extended these national activities to an international stage (ICECDogs 2024). In recent years, there has been more coordinated movement towards improvement of purebred cat welfare, for example, a request to breeding registries to consider mandatory genetic testing (Lyons 2024), publication of an Animal Welfare Committee report on breeding cats in the UK (DEFRA 2024) and formation of the World Small Animal Veterinary Association (WSAVA) Hereditary Disease Committee (WSAVA undated). However, coordinated national and international collaborative activities to protect feline welfare have historically lagged far behind that of dogs. This could be turned to an advantage, where feline advocates can learn lessons from both successful and failed interventions to alleviate the health and welfare crisis enveloping purebred dogs and use this to prevent the same welfare harms from happening to cats. Advocates for feline welfare can replicate and improve upon those national and international bodies that are most effective in protecting canine health and welfare to create similar bodies to protect cats.

## Conclusion

With an increasing proportion of cats in the UK being reported as purebred, protecting the welfare of purebred cats has never been more vital. The evidence reviewed in the current study highlights that purebred cat welfare repeatedly falls outside of the legislative boundaries set out by the Animal Welfare Acts. While many literature gaps have been identified, there are several areas with good evidence of welfare compromise which could, and should, be addressed immediately, including folded ears and severe brachycephalism. Assessment of pain in cats bred without tails and dwarf cats has been overlooked and should be a priority area for new research. However, given that cats evolved over many millions of years to show a consistent feline phenotype worldwide that maximised their survival, and that cats evolved natural behaviours aligned to these phenotypes, it would appear scientifically reasonable to require prior evidence of welfare neutrality for any new phenotypes deliberately selected for introduction to cats before such new body shapes were considered legally, ethically or morally acceptable in future cats. Absence of evidence should not be blindly accepted as evidence of absence.

Alongside data on the UK prevalence of traits that impede welfare across purebred cats, aspects where research is lacking include breeding conditions for purebred cats, impact of morphological changes on social behaviour of cats, evidence on the breeding and keeping of hybrid cats, the uptake and impact of genetic testing and evidence on human motivations to own cats with extreme conformation. In the modern domestic setting where many cats now live, the love of humans for the spectacular in their cat and the consequent collective breed selection choices presents a very real and growing threat to cat welfare.

## Supporting information

Roberts et al. supplementary material Roberts et al. supplementary material

## Supplementary material

The supplementary material for this article can be found at http://doi.org/10.1017/awf.2025.10051.

## References

[r1] AAFP 2017 *AAFP Position Statement Hybrid Cats.* https://catvets.com/wp-content/uploads/2024/01/2017-HybridCats-Statement.pdf (accessed Jan 2025).

[r2] Abitbol M, Dufaure de Citres C, Rudd Garces G, Lühken G, Lyons LA and Gache V 2024 Different founding effects underlie dominant blue eyes (DBE) in the domestic cat. Animals 14: 1845. 10.3390/ani14131845 38997957 PMC11240321

[r3] Åhman SE and Bergström KE 2009 Cutaneous carriage of Malassezia species in healthy and seborrhoeic Sphynx cats and a comparison to carriage in Devon Rex cats. Journal of Feline Medicine and Surgery 11: 970–976. 10.1016/j.jfms.2009.04.011 19559635 PMC11318777

[r4] Ahmed J 2024 *Warning as ‘exaggerated’ hairless ‘bullycats’ being bred in UK despite serious health issues.* https://www.independent.co.uk/news/uk/home-news/hairless-xl-bully-cats-rspca-health-warning-b2597230.html (accessed Jan 2025).

[r5] Albasan H, Osborne CA, Lulich JP and Lekcharoensuk C 2012 Risk factors for urate uroliths in cats. Journal of the American Veterinary Medical Association 240: 842–847. 10.2460/javma.240.7.842 22443437

[r6] Allan GS 2000 Radiographic features of feline joint diseases. The Veterinary Clinics of North America Small Animal Practice 30: 281–302. 10768235

[r7] Amat M, de la Torre JLR, Fatjó J, Mariotti VM, Van Wijk S and Manteca X 2009 Potential risk factors associated with feline behaviour problems. Applied Animal Behaviour Science 121: 134–139. 10.1016/j.applanim.2009.09.012

[r8] Anagrius KL, Dimopoulou M, Moe AN, Petterson A and Ljungvall I 2021 Facial conformation characteristics in Persian and Exotic Shorthair cats. Journal of Feline Medicine and Surgery 23: 1089–1097. 10.1177/1098612x21997631 33655782 PMC8637354

[r9] Anderson H, Davison S, Lytle KM, Honkanen L, Freyer J, Mathlin J, Kyöstilä K, Inman L, Louviere A, Chodroff Foran R, Forman OP, Lohi H and Donner J 2022 Genetic epidemiology of blood type, disease and trait variants, and genome-wide genetic diversity in over 11,000 domestic cats. PLoS Genetics 16: e1009804. 10.1371/journal.pgen.1009804 PMC9202916 35709088

[r10] Anderson LM, Fox DB, Chesney KL, Coates JR, Torres BT and Lyons LA 2021 Skeletal manifestations of heritable disproportionate dwarfism in cats as determined by radiography and magnetic resonance imaging. Veterinary and Comparative Orthopaedics and Traumatology 34: 327–337. 10.1055/s-0041-1730355 34082456 PMC10207382

[r11] Åsbjer E, Hedhammar Å and Engdahl K 2024 Awareness, experiences, and opinions by owners, breeders, show judges, and veterinarians on canine Brachycephalic Obstructive Airway Syndrome (BOAS). Canine Medicine and Genetics 11: 3. 10.1186/s40575-024-00137-4 38459530 PMC10924362

[r12] AWF and RSPCA 2018 *The Puppy Contract.* https://puppycontract.org.uk/sites/default/files/2018-10/The%20Puppy%20Contract.pdf (accessed Jan 2025).

[r13] Bamber RC 1933 Correlation between white coat colour, blue eyes and deafness in cats. Journal of Genetics 27: 407–413. 10.1007/bf02981752

[r14] Barcelos AM, Kargas N and Mills D 2024 The effects of dog behavioural problems on owner well-being: A review of the literature and future directions. Pets 1(1): 53–69. 10.3390/pets1010007

[r15] Beban A, Korson C, Reid J, Procter J, Halley J and Mackenzie K 2024 Building a Place-based Social Licence to Operate. AgResearch, New Zealand. https://agresearch.figshare.com/articles/report/Building_a_Place-based_Social_Licence_to_Operate/26001745?file=46944826 (accessed 17 October 2025).

[r16] Berteselli GV, Palestrini C, Scarpazza F, Barbieri S, Prato-Previde E and Cannas S 2023 Flat-faced or non-flat-faced cats? That is the question. Animals 13. 10.3390/ani13020206 PMC9854927 36670746

[r17] Bijsmans ES, Jepson RE, Syme HM, Elliott J and Niessen SJM 2016 Psychometric validation of a general health Quality of Life tool for cats used to compare healthy cats and cats with chronic kidney disease. Journal of Veterinary Internal Medicine 30: 183–191. 10.1111/jvim.13656 26567089 PMC4913638

[r18] Biller DS, DiBartola SP, Eaton KA, Pflueger S, Wellman ML and Radin MJ 1996 Inheritance of polycystic kidney disease in Persian cats. Journal of Heredity 87: 1–5. 10.1093/oxfordjournals.jhered.a022945 8742815

[r19] Binns S and Dawson S 1995 Feline infectious upper respiratory disease. *In* Practice 17: 458–461. 10.1136/inpract.17.10.458

[r20] Bradshaw J 2018 Normal feline behaviour:… and why problem behaviours develop. Journal of Feline Medicine and Surgery 20: 411–421. 10.1177/1098612x18771203 29706092 PMC11395290

[r21] Buckley RM, Davis BW, Brashear WA, Farias FHG, Kuroki K, Graves T, Hillier LW, Kremitzki M, Li G, Middleton RP, Minx P, Tomlinson C, Lyons LA, Murphy WJ and Warren WC 2020 A new domestic cat genome assembly based on long sequence reads empowers feline genomic medicine and identifies a novel gene for dwarfism. PLoS Genetics 16: e1008926. 10.1371/journal.pgen.1008926 33090996 PMC7581003

[r22] Buffington CAT 2002 External and internal influences on disease risk in cats. Journal of the American Veterinary Medical Association 220: 994–1002. 10.2460/javma.2002.220.994 12420776

[r23] BVA 2018 *Policy Statement Brachycephalic dogs.* https://www.bva.co.uk/media/1183/bva-position-on-brachycephalic-dogs-full.pdf (accessed Jan 2025).

[r24] BWG 2024 *The Brachycephalic Working Group.* The Brachycephalic Working Group. http://www.ukbwg.org.uk/ (Accessed Jan 2024).

[r25] Cafazzo S and Natoli E 2009 The social function of tail up in the domestic cat (*Felis silvestris catus*). Behavioural Processes 80: 60–66. 10.1016/j.beproc.2008.09.008 18930121

[r26] Campbell GR, Arnott ER, Graham C, Niel L, Ward MP and Ma G 2024 Impact of early socialisation in foster care on kitten behaviour. Applied Animal Behaviour Science: 106306. 10.1016/j.applanim.2024.106306

[r27] Cannas S, Alessi S, Scarpazza F and Palestrini C 2023 Assessment of cats’ behavior during a cat show. Journal of Veterinary Behavior 62: 53–63. 10.1016/j.jveb.2023.02.007

[r28] Cannon MJ, Barr FJ, Rudorf H, Bradley KJ, Gruffydd‐Jones TJ and MacKay AD 2001 Prevalence of polycystic kidney disease in Persian cats in the United Kingdom. Veterinary Record 149: 409–411. 10.1136/vr.149.14.409 11678212

[r29] Casal ML 2022 Feline Fertility Consequences of inbreeding and implications for reproductive fitness. Journal of Feline Medicine and Surgery 24: 847–852. 10.1177/1098612x221118755 36002141 PMC10812227

[r30] Casamian‐Sorrosal D, Chong SK, Fonfara S and Helps C 2014 Prevalence and demographics of the MYBPC3‐mutations in Ragdolls and Maine Coons in the British Isles. Journal of Small Animal Practice 55: 269–273. 10.1111/jsap.12201 24602043

[r31] Casey RA and Bradshaw JWS 2008 The effects of additional socialisation for kittens in a rescue centre on their behaviour and suitability as a pet. Applied Animal Behaviour Science 114: 196–205. 10.1016/j.applanim.2008.01.003

[r32] Cats Protection 2023 *Cats for clicks – social media driving worrying trend for pedigree cats, says Cats Protection.* https://www.cats.org.uk/mediacentre/pressreleases/cats-for-clicks-social-media-driving-worrying-trend-for-pedigree-cats-says-cats-protection (accessed July 2025).

[r33] Cats Protection 2024 *Cats and Their Stats CATS Report 2024.* https://www.cats.org.uk/about-cp/cats-report (accessed Feb 2025).

[r34] Cats Protection (undated) Getting a pedigree cat. https://www.cats.org.uk/help-and-advice/getting-a-cat/getting-a-pedigree-cat (accessed Jan 2025).

[r35] Černá P, Pugalendhi SJ, Shaw DJ and Gunn-Moore DA 2024 Feline dystocia and kitten mortality up to 12 weeks in pedigree cats. Journal of Feline Medicine and Surgery 26: 1098612X241284766. 10.1177/1098612X241284766 PMC11632851 39656270

[r36] Chang J, Jung J, Oh S, Lee S, Kim G, Kim H, Kweon O, Yoon J and Choi M 2007 Osteochondrodysplasia in three Scottish Fold cats. Journal of Veterinary Science 8: 307–309. 10.4142/jvs.2007.8.3.307 17679781 PMC2868141

[r37] Coggins SJ, Norris JM, Malik R, Govendir M, Hall EJ, Kimble B and Thompson MF 2023 Outcomes of treatment of cats with feline infectious peritonitis using parenterally administered remdesivir, with or without transition to orally administered GS-441524. Journal of Veterinary Internal Medicine 37: 1772–1783. 10.1111/jvim.16803 37439383 PMC10473006

[r38] Deforest ME and Basrur PK 1979 Malformations and the Manx syndrome in cats. The Canadian Veterinary Journal 20: 304. 393376 PMC1789620

[r39] DEFRA 2024 *Opinion on the welfare implications of current and emergent feline breeding practices.* https://www.gov.uk/government/publications/opinion-on-cat-breeding-practices/opinion-on-the-welfare-implications-of-current-and-emergent-feline-breeding-practices#introduction-and-scope (accessed Aug 2025).

[r40] Delgado MM and Reevy GM 2018 Development and psychometric evaluation of the cat care and needs scale (CCANS). Anthrozoös 31: 89–100. 10.1080/08927936.2018.1406203

[r41] Demir A 2024 Assessment of eye disorders in brachycephalic cat breeds-a retrospective study of 328 cases (2018-2022). Veterinarski Arhiv 94: 237–254. 10.24099/vet.arhiv.1891

[r42] Deputte BL, Jumelet E, Gilbert C and Titeux E 2021 Heads and tails: An analysis of visual signals in cats, felis catus. Animals 11. 10.3390/ani11092752 PMC8469685 34573718

[r43] De Santis-Kerr AC, Raghavan M, Glickman NW, Caldanaro RJ, Moore GE, Lewis HB, Schantz PM and Glickman LT 2006 Prevalence and risk factors for Giardia and coccidia species of pet cats in 2003-2004. Journal of Feline Medicine and Surgery 8: 292–301. 10.1016/j.jfms.2006.02.005 16678461 PMC10822243

[r44] Doit H, Dean RS, Duz M and Brennan ML 2021 A systematic review of the quality of life assessment tools for cats in the published literature. The Veterinary Journal 272: 105658. 33941335 10.1016/j.tvjl.2021.105658

[r45] Eagan BH, Gordon E and Fraser D 2021 The effect of animal shelter sound on cat behaviour and welfare. Animal Welfare 30: 431–440. 10.7120/09627286.30.4.006

[r46] Egenvall A, Bonnett BN, Häggström J, Ström Holst B, Möller L and Nødtvedt A 2010 Morbidity of insured Swedish cats during 1999-2006 by age, breed, sex, and diagnosis. Journal of Feline Medicine and Surgery 12: 948–959. 10.1016/j.jfms.2010.08.008 21055987 PMC11135553

[r47] Ekstrand C and Linde‐Forsberg C 1994 Dystocia in the cat: a retrospective study of 155 cases. Journal of Small Animal Practice 35: 459–464. 10.1111/j.1748-5827.1994.tb03951.x

[r48] Ellis Sarah LH, Rodan I, Carney HC, Heath S, Rochlitz I, Shearburn LD, Sundahl E, and Westropp JL 2013 AAFP and ISFM feline environmental needs guidelines. Journal of Feline Medicine and Surgery 15: 219–230. 10.1177/1098612x13477537 23422366 PMC11383066

[r49] European Union 2025 *Proposal for a regulation of the European Parliament and of the Council on the welfare of dogs and cats and their traceability.* https://eur-lex.europa.eu/legal-content/EN/TXT/?uri=COM:2023:769:FIN (accessed Aug 2025).

[r50] Evangelista MC, Watanabe R, Leung VSY, Monteiro BP, O’Toole E, Pang DSJ and Steagall PV 2019 Facial expressions of pain in cats: the development and validation of a Feline Grimace Scale. Scientific Reports 9: 19128. 10.1038/s41598-019-55693-8 31836868 PMC6911058

[r51] Farnworth MJ, Chen R, Packer RMA, Caney SMA and Gunn-Moore DA 2016 Flat feline faces: Is brachycephaly associated with respiratory abnormalities in the domestic cat (*Felis catus*)? *PLoS ONE* 11. 10.1371/journal.pone.0161777 PMC5004878 27574987

[r52] Farnworth MJ, Packer RMA, Sordo L, Chen R, Caney SMA and Gunn-Moore DA 2018 In the eye of the beholder: Owner preferences for variations in cats’ appearances with specific focus on skull morphology. Animals 8: 30. 10.3390/ani8020030 29461472 PMC5836038

[r53] FIFe 2025 *FIFe breeding and registration rules.* https://fifeweb.org/app/uploads/2023/11/br_reg_en.pdf (accessed Feb 2025).

[r54] FIFe (undated) *Breeds.* https://fifeweb.org/cats/breeds/ (accessed Feb 2025).

[r55] Finch NC, Syme HM and Elliott J 2016 Risk factors for development of chronic kidney disease in cats. Journal of Veterinary Internal Medicine 30: 602–610. 10.1111/jvim.13917 26948860 PMC4864943

[r56] Finka LR and Foreman-Worsley R 2022 Are multi-cat homes more stressful? A critical review of the evidence associated with cat group size and wellbeing. Journal of Feline Medicine and Surgery 24: 65–76. 10.1177/1098612x211013741 34037488 PMC8807997

[r57] Finka LR, Luna SP, Brondani JT, Tzimiropoulos Y, McDonagh J, Farnworth MJ, Ruta M and Mills DS 2019 Geometric morphometrics for the study of facial expressions in non-human animals, using the domestic cat as an exemplar. Scientific reports 9: 9883. 10.1038/s41598-019-46330-5 31285531 PMC6614427

[r58] Foreman-Worsley R and Farnworth MJ 2019 A systematic review of social and environmental factors and their implications for indoor cat welfare. Applied Animal Behaviour Science 220: 104841. 10.1016/j.applanim.2019.104841

[r59] Foreman-Worsley R, Finka LR, Ward SJ and Farnworth MJ 2021 Indoors or outdoors? An international exploration of owner demographics and decision making associated with lifestyle of pet cats. Animals 11: 253. 10.3390/ani11020253 33498511 PMC7909512

[r60] Freeman LM, Rodenberg C, Narayanan A, Olding J, Gooding MA and Koochaki PE 2016 Development and initial validation of the Cat HEalth and Wellbeing (CHEW) Questionnaire: a generic health-related quality of life instrument for cats. Journal of Feline Medicine and Surgery 18: 689–701. 10.1177/1098612X16657386 27562979 PMC11148897

[r61] Freeman LM, Rush JE, Oyama MA, MacDonald KA, Cunningham SM, Bulmer B, MacGregor JM, Laste NJ, Malakoff RL and Hall DJ 2012 Development and evaluation of a questionnaire for assessment of health-related quality of life in cats with cardiac disease. Journal of the American Veterinary Medical Association 240: 1188–1193. 10.2460/javma.240.10.1188 22559108

[r62] Gandolfi B, Alamri S, Darby WG, Adhikari B, Lattimer JC, Malik R, Wade CM, Lyons LA, Cheng J, Bateman JF, McIntyre P, Lamandé SR and Haase B 2016 A dominant TRPV4 variant underlies osteochondrodysplasia in Scottish fold cats. Osteoarthritis and Cartilage 24: 1441–1450. 10.1016/j.joca.2016.03.019 27063440

[r63] GCCF 2017 *The GCCF Registration policy for Bengal cats.* https://www.gccfcats.org/wp-content/uploads/2021/10/Bengal-RP-2017.pdf (accessed Jan 2025).

[r64] GCCF 2024 *Registered cats by breed.* https://www.gccfcats.org/wp-content/uploads/2025/01/Registered-Cats-By-Breed-2023-2024.pdf (accessed Jan 2025).

[r65] GCCF (undated a) *Cat Breeds.* https://www.gccfcats.org/getting-a-cat/choosing/cat-breeds/ (accessed Jan 2025).

[r66] GCCF (undated b) *Welfare.* https://www.gccfcats.org/welfare/ (accessed Jan 2025).

[r67] GCCF (undated c) *British.* https://www.gccfcats.org/getting-a-cat/choosing/cat-breeds/british/ (accessed Jan 2025).

[r68] GCCF (undated d) *Sphynx care.* https://www.gccfcats.org/getting-a-cat/choosing/cat-breeds/sphynx/ (accessed Jan 2025).

[r69] GCCF (undated e) *Genetic testing.* https://www.gccfcats.org/breeding-cats/new-to-breeding/testing/gene-testing/ (accessed Jul 2025).

[r70] GCCF (undated f) *Ragdoll.* https://www.gccfcats.org/getting-a-cat/choosing/cat-breeds/ragdoll/ (accessed Jan 2025).

[r71] Gershony LC, Penedo MCT, Davis BW, Murphy WJ, Helps CR and Lyons LA 2014 Who’s behind that mask and cape? the Asian leopard cat’s Agouti (ASIP) allele likely affects coat colour phenotype in the Bengal cat breed. Animal Genetics 45: 893–897. 10.1111/age.12206 25143047 PMC4211939

[r72] Glanville C, Hampton JO and Sandøe P 2025 Calling a trade-off a trade-off in arguments for cat confinement. Animal Welfare 34: e65. 10.1017/awf.2025.10041 41158302 PMC12554810

[r73] Gleason HE, Phillips H and McCoy AM 2023 Influence of feline brachycephaly on respiratory, gastrointestinal, sleep, and activity abnormalities. Veterinary Surgery 52: 435–445. 10.1111/vsu.13931 36582029

[r74] Go Compare 2024 *Cat Insurance.* https://www.gocompare.com/pet-insurance/cat-insurance/ (accessed Feb 2025).

[r75] Goericke-Pesch S and Packeiser E-M 2022 Reproductive management in catteries: optimising health and wellbeing through veterinarian-breeder collaboration. Journal of Feline Medicine and Surgery 24: 881–904. 10.1177/1098612x221118760 36002135 PMC10812226

[r76] Gough A, Thomas A and O’Neill D 2018 Breed predispositions to disease in dogs and cats. John Wiley & Sons: London, UK.

[r77] Granström S, Nyberg Godiksen MT, Christiansen M, Pipper CB, Willesen JT and Koch J 2011 Prevalence of hypertrophic cardiomyopathy in a cohort of British Shorthair Cats in Denmark. Journal of Veterinary Internal Medicine 25: 866–871. 10.1111/j.1939-1676.2011.0751.x 21736622

[r78] Grigg EK and Kogan LR 2019 Owners’ attitudes, knowledge, and care practices: Exploring the implications for domestic cat behavior and welfare in the home. Animals 9. 10.3390/ani9110978 PMC6912669 31731680

[r79] Gunn-Moore DA and Thrusfield M V 1995 Feline dystocia: prevalence, and association with cranial conformation and breed. The Veterinary Record 136: 350–353. 7610538 10.1136/vr.136.14.350

[r80] Häggström J, Luis Fuentes V and Wess G 2015 December 1 Screening for hypertrophic cardiomyopathy in cats. Elsevier BV: The Netherlands. 10.1016/j.jvc.2015.07.003 26776573

[r81] Hagman R, Ström Holst B, Möller L and Egenvall A 2014 Incidence of pyometra in Swedish insured cats. Theriogenology 82: 114–120. 10.1016/j.theriogenology.2014.03.007 24726694

[r82] Hamelin A, Begon D, Conchou F, Fusellier M and Abitbol M 2017 Clinical characterisation of polydactyly in Maine Coon cats. Journal of Feline Medicine and Surgery 19: 382–393. 10.1177/1098612X16628920 26862149 PMC11119636

[r83] Herron ME 2010 Advances in understanding and treatment of feline inappropriate elimination. Topics in Companion Animal Medicine 25: 195–202. 10.1053/j.tcam.2010.09.005 21147472

[r84] Herron ME and Buffington CAT 2010 Environmental enrichment for indoor cats. *Compendium (Yardley, PA)* 32: E4. PMC3922041 21882164

[r85] Hodgman SFJ 1963 Abnormalities and defects in pedigree dogs–I. An investigation into the existence of abnormalities in pedigree dogs in the British Isles. Journal of Small Animal Practice 4: 447–456. 10.1111/j.1748-5827.1963.tb01301.x

[r86] Horwitz DF 1997 Behavioral and environmental factors associated with elimination behavior problems in cats: a retrospective study. Applied Animal Behaviour Science 52: 129–137. 10.1016/s0168-1591(96)01073-8

[r87] ICECDogs 2024 *International Collaborative on Extreme Conformations in Dogs.* International Partnership for Dogs. https://www.icecdogs.com/ (accessed 8 October 2024).

[r88] International Cat Care 2023 *Urging advertisers to ban the use of cat breeds with extreme conformation.* https://icatcare.org/news/icatcare-joins-fecava-fve-and-uevp-to-urge-advertisers-to-ban-the-use-of-cat-breeds-with-extreme-conformation (accessed Jul 2025).

[r89] International Cat Care (undated) *Position statement on the breeding of pedigree cats.* https://icatcare.org/position-statement.s/position-statement-on-the-breeding-of-pedigree-cats (accessed Feb 2025).

[r90] Karsh EB and Turner DC 1998 The human–cat relationship In: Turner DC and Bateson P (eds) The Domestic Cat: The Biology of Its Behaviour pp 67–68. Cambridge University Press: Cambridge, UK.

[r91] Keller GG, Reed AL, Lattimer JC and Corley EA 1999 Hip dysplasia: a feline population study. Veterinary Radiology & Ultrasound 40: 460–464. 10.1111/j.1740-8261.1999.tb00375.x 10528838

[r92] Kihara S, Aikawa T, Miyazaki Y, Nishimura M and Muyama H 2024 Thoracic Vertebral Canal Stenosis and Vertebral Instability in a Young Minuet Cat. Journal of the American Animal Hospital Association 60: 81–86. 10.5326/jaaha-ms-7403 38394696

[r93] Kittleson MD and Côté E 2021 The feline cardiomyopathies: 2. Hypertrophic cardiomyopathy. Journal of Feline Medicine and Surgery 23: 1028–1051. 34693811 10.1177/1098612X211020162 PMC8642168

[r94] Kittleson MD, Meurs KM, Munro MJ, Kittleson JA, Liu S-K, Pion PD and Towbin JA 1999 Familial hypertrophic cardiomyopathy in maine coon cats: an animal model of human disease. Circulation 99: 3172–3180. 10.1177/1098612x211020162 10377082

[r95] Klein-Richers U, Hartmann K, Hofmann-Lehmann R, Unterer S, Bergmann M, Rieger A, Leutenegger C, Pantchev N, Balzer J and Felten S 2020 Prevalence of feline coronavirus shedding in German catteries and associated risk factors. Viruses 12: 1000. 10.3390/v12091000 32911718 PMC7551668

[r96] Köhler I, Ballhausen BD, Stockhaus C, Hartmann K and Wehner A 2016 Prevalence of and risk factors for feline hyperthyroidism among a clinic population in Southern Germany. Tierärztliche Prax. Kleintiere 44: 149–157. 10.15654/tpk-150590 26902958

[r97] Künzel W, Breit S and Oppel M 2003 Morphometric investigations of breed‐specific features in feline skulls and considerations on their functional implications. Anatomia, Histologia, Embryologia 32: 218–223. 10.1046/j.1439-0264.2003.00448.x 12919072

[r98] LAGECDogs 2024 *Legal Advisory Group on Extreme Conformation in Dogs.* A-LAW UK Centre for Animal Law. https://www.alaw.org.uk/companion-animals/extreme-dog-conformation/ (accessed 10 January 2024).

[r99] Langford Vets (undated) *Cat Genetic Diseases and Breed.* https://www.langfordvets.co.uk/diagnostic-laboratories/cat-genetic-testing/cat-genetic-diseases-and-breeds/ (accessed Feb 2025).

[r100] Langley‐Hobbs S 2023 Update on diagnosing and managing osteoarthritis in cats. *In* Practice 45: 594–608. 10.1002/inpr.380

[r101] Lawson GT, Langford FM and Harvey AM 2020 The environmental needs of many Australian pet cats are not being met. Journal of Feline Medicine and Surgery 22: 898–906. 10.1177/1098612X19890189 31841057 PMC10814394

[r102] Lederer R, Rand JS, Jonsson NN, Hughes IP and Morton JM 2009 Frequency of feline diabetes mellitus and breed predisposition in domestic cats in Australia. Veterinary Journal 179: 254–258. 10.1016/j.tvjl.2007.09.019 18155627

[r103] Leipold H, Huston K, Blauch B and Guffy MM 1974 Congenital defects of the caudal vertebral column and spinal cord in Manx cats. Journal of the American Veterinary Medical Association 164: 520–523. 10.2460/javma.1974.164.05.520 4813411

[r104] Lipinski MJ, Froenicke L, Baysac KC, Billings NC, Leutenegger CM, Levy AM, Longeri M, Niini T, Ozpinar H, Slater MR, Pedersen NC and Lyons LA 2008 The ascent of cat breeds: Genetic evaluations of breeds and worldwide random-bred populations. Genomics 91: 12–21. 10.1016/j.ygeno.2007.10.009 18060738 PMC2267438

[r105] Loder RT and Todhunter RJ 2018 Demographics of hip dysplasia in the Maine Coon cat. Journal of Feline Medicine and Surgery 20: 302–307. 10.1177/1098612x17705554 28430011 PMC11129213

[r106] Lue TW, Pantenburg DP and Crawford PM 2008 Impact of the owner-pet and client-veterinarian bond on the care that pets receive. Journal of the American Veterinary Medical Association 232: 531–540. 10.2460/javma.232.4.531 18279086

[r107] Lund E 2012 Epidemiology of periodontal disease in older cats. Veterinary Focus 22: 23–24.

[r108] Lynch S, Savary-Bataille K, Leeuw B and Argyle DJ 2011 Development of a questionnaire assessing health-related quality-of-life in dogs and cats with cancer. Veterinary and Comparative Oncology 9: 172–182. 10.1111/j.1476-5829.2010.00244.x 21848620

[r109] Lyons LA 2024 Genetic testing: practical dos and don’ts for cats. Journal of Feline Medicine and Surgery 26: 10.1177/1098612X241303603 DOI? PMC11626677 39648935

[r110] Lyons LA, Biller DS, Erdman CA, Lipinski MJ, Young AE, Roe BA, Qin B and Grahn RA 2004 Feline polycystic kidney disease mutation identified in PKD1. Journal of the American Society of Nephrology 15: 2548–2555. 10.1097/01.ASN.0000141776.38527.BB 15466259

[r111] Lyons LA, Fox DB, Chesney KL, Britt LG, Buckley RM, Coates JR, Gandolfi B, Grahn RA, Hamilton MJ and Middleton JR 2019 Localization of a feline autosomal dominant dwarfism locus: a novel model of chondrodysplasia. BioRxiv: 687210. 10.1101/687210

[r112] Machado D de S, Gonçalves L da S, Vicentini RR, Ceballos MC and Sant’Anna AC 2020 Beloved whiskers: management type, care practices and connections to welfare in domestic cats. Animals 10: 2308. 10.3390/ani10122308 33291476 PMC7762120

[r113] Malik R, Allan GS, Howlett CR, Thompson DE, James G, McWhirter C and Kendall K 1999 Osteochondrodysplasia in Scottish Fold cats. Australian Veterinary Journal 77: 85–92. 10.1111/j.1751-0813.1999.tb11672.x 10078353

[r114] Mariti C, Guerrini F, Vallini V, Bowen JE, Fatjó J, Diverio S, Sighieri C and Gazzano A 2017 The perception of cat stress by Italian owners. Journal of Veterinary Behavior 20: 74–81. 10.1016/j.jveb.2017.04.002

[r115] Maron BJ 2018 Clinical course and management of hypertrophic cardiomyopathy. New England Journal of Medicine 379: 655–668. 10.1056/nejmc1812159 30110588

[r116] Marsh DM and Hanlon TJ 2007 Seeing what we want to see: Confirmation bias in animal behavior research. Ethology 113: 1089–1098. 10.1111/j.1439-0310.2007.01406.x

[r117] Martin AH 1971 A congenital defect in the spinal cord of the Manx cat. Veterinary Pathology 8: 232–238. 10.1177/030098587100800305 4950726

[r118] McCann TM, Simpson KE, Shaw DJ, Butt JA and Gunn-Moore D 2007 Feline diabetes mellitus in the UK: the prevalence within an insured cat population and a questionnaire-based putative risk factor analysis. Journal of Feline Medicine and Surgery 9: 289–299. 10.1016/j.jfms.2007.02.001 17392005 PMC10822632

[r119] McCune S 1995 The impact of paternity and early socialisation on the development of cats’ behaviour to people and novel objects. Applied Animal Behaviour Science 45: 109–124. 10.1016/0168-1591(95)00603-p

[r120] McEntee A 2021 *What you need to know about pedigree cat insurance.* https://www.comparethemarket.com/pet-insurance/content/pedigree-cat-insurance/ (accessed Feb 2025).

[r121] McGrath JI, Zhang W, Hollar R, Collings A, Powell R, Foale RD, Thurley N, Brockman JA, Mellanby RJ, Gunn-Moore DA and Schoenebeck JJ 2021 More than a moggy; a population genetics analysis of the United Kingdom’s non-pedigree cats. Genes 12. 10.3390/genes12101619 PMC8535647 34681013

[r122] McGreevy PD and Nicholas FW 1999 Some practical solutions to welfare problems in dog breeding. Animal Welfare 8: 329–341. 10.1017/s0962728600021965

[r123] Mestrinho LA, Louro JM, Gordo IS, Niza MMRE, Requicha JF, Force JG and Gawor JP 2018 Oral and dental anomalies in purebred, brachycephalic Persian and Exotic cats. Journal of the American Veterinary Medical Association 253: 66–72. 10.2460/javma.253.1.66 29911947

[r124] Meurs KM, Norgard MM, Kuan M, Haggstrom J and Kittleson M 2009 Analysis of 8 sarcomeric candidate genes for feline hypertrophic cardiomyopathy mutations in cats with hypertrophic cardiomyopathy. Journal of Veterinary Internal Medicine 23: 840–843. 10.1111/j.1939-1676.2009.0341.x 19566849

[r125] Mills DS, Demontigny-Bédard I, Gruen M, Klinck MP, McPeake KJ, Barcelos AM, Hewison L, Van Haevermaet H, Denenberg S and Hauser H 2020 Pain and problem behavior in cats and dogs. Animals 10: 318. 10.3390/ani10020318 32085528 PMC7071134

[r126] Mitze S, Barrs VR, Beatty JA, Hobi S and Bęczkowski PM 2022 Brachycephalic obstructive airway syndrome: much more than a surgical problem. Veterinary Quarterly 42: 213–223. 10.1080/01652176.2022.2145621 36342210 PMC9673814

[r127] Monteiro CLB, Campos AIM, Madeira VLH, Silva HVR, Freire LMP, Pinto JN, De Souza LP and Da Silva LDM 2013 Pelvic differences between brachycephalic and mesaticephalic cats and indirect pelvimetry assessment. Veterinary Record 172: 16. 10.1136/vr.100859 23118051

[r128] Morel E, Malineau L, Venet C, Gaillard V and Péron F 2024 Prioritization of appearance over health and temperament is detrimental to the welfare of purebred dogs and cats. Animals 14. 10.3390/ani14071003 PMC11011023 38612242

[r129] Morris D 1999 Cat Breeds of the World. A Complete Illustrated Encyclopedia. Viking Penguin: London, UK.

[r130] New Zealand Government 2024 *Step-by-step guide to bringing cats and dogs to NZ.* https://www.mpi.govt.nz/bring-send-to-nz/pets-travelling-to-nz/bringing-cats-and-dogs-to-nz/step-by-step-guide-to-bringing-cats-and-dogs-to-nz/ (accessed October 2025).

[r131] Niessen SJM, Powney S, Guitian J, Niessen APM, Pion PD, Shaw JAM and Church DB 2010 Evaluation of a quality-of-life tool for cats with diabetes mellitus. Journal of Veterinary Internal Medicine 24: 1098–1105. 10.1111/j.1939-1676.2010.0579.x 20707839

[r132] Nicholas FW, Tammen I, and Sydney Informatics Hub. 2025 *Online Mendelian Inheritance in Animals (OMIA) (cat).* 10.25910/2AMR-PV70 (accessed October 2025).

[r133] Noble CE, Wiseman-Orr LM, Scott ME, Nolan AM and Reid J 2019 Development, initial validation and reliability testing of a web-based, generic feline health-related quality-of-life instrument. Journal of Feline Medicine and Surgery 21: 84–94. 10.1177/1098612X18758176 29463202 PMC10814614

[r134] Noli C, Borio S, Varina A and Schievano C 2016 Development and validation of a questionnaire to evaluate the Quality of Life of cats with skin disease and their owners, and its use in 185 cats with skin disease. Veterinary Dermatology 27: 247–258. 10.1111/vde.12341 27292136

[r135] O’Halloran C, Tørnqvist-Johnsen C, Woods G, Mitchell J, Reed N, Burr P, Gascoyne-Binzi D, Wegg M, Beardall S, Hope J and Gunn-Moore D 2021 Feline tuberculosis caused by Mycobacterium bovis infection of domestic UK cats associated with feeding a commercial raw food diet. Transboundary and Emerging Diseases 68: 2308–2320. 10.1111/tbed.13889 33091235

[r136] Öhlund M, Fall T, Ström Holst B, Hansson-Hamlin H, Bonnett B and Egenvall A 2015 Incidence of Diabetes Mellitus in Insured Swedish Cats in Relation to Age, Breed and Sex. Journal of Veterinary Internal Medicine 29: 1342–1347. 10.1111/jvim.13584 26179258 PMC4858030

[r137] O’Neill DG, Blenkarn A, Brodbelt DC, Church DB and Freeman A 2023a Periodontal disease in cats under primary veterinary care in the UK: frequency and risk factors. Journal of Feline Medicine and Surgery 25: 1098612X231158154. 10.1177/1098612x231158154 PMC10812011 36912667

[r138] O’Neill DG, Church DB, McGreevy PD, Thomson PC and Brodbelt DC 2015 Longevity and mortality of cats attending primary care veterinary practices in England. Journal of Feline Medicine and Surgery 17: 125–133. 10.1177/1098612x14536176 24925771 PMC10816413

[r139] O’Neill DG, Gostelow R, Orme C, Church DB, Niessen SJM, Verheyen K and Brodbelt DC 2016 Epidemiology of diabetes mellitus among 193,435 cats attending primary-care veterinary practices in England. Journal of Veterinary Internal Medicine 30: 964–972. 10.1111/jvim.14365 27353396 PMC5094533

[r140] O’Neill DG, McMillan KM, Church DB and Brodbelt DC 2023b Dog breeds and conformations in the UK in 2019: VetCompass canine demography and some consequent welfare implications. PLOS One 18: e0288081. 10.1371/journal.pone.0288081 37494312 PMC10370710

[r141] Overley B, Shofer FS, Goldschmidt MH, Sherer D and Sorenmo KU 2005 Association between ovarihysterectomy and feline mammary carcinoma. Journal of Veterinary Internal Medicine 19: 560–563. 10.1111/j.1939-1676.2005.tb02727.x 16095174

[r142] Packer RMA, Hendricks A and Burn CC 2012 Do dog owners perceive the clinical signs related to conformational inherited disorders as ‘normal’for the breed? A potential constraint to improving canine welfare. Animal Welfare 21: 81–93. 10.7120/096272812X13345905673809

[r143] Packer RMA, Murphy D and Farnworth MJ 2017 Purchasing popular purebreds: investigating the influence of breed-type on the pre-purchase motivations and behaviour of dog owners. Animal Welfare 26: 191–201. 10.7120/09627286.26.2.191

[r144] Packer R and O’Neill D 2021 Health and Welfare of Brachycephalic (Flat-Faced) Companion Animals: A Complete Guide for Veterinary and Animal Professionals. CRC: Boca Raton, USA.

[r145] Packer RMA, Wade A and Neufuss J 2024 Nothing could put me off: assessing the prevalence and risk factors for perceptual barriers to improving the welfare of brachycephalic dogs. Pets 1: 458–484. 10.3390/pets1030032

[r146] PDSA (undated a) *Sphynx.* https://www.pdsa.org.uk/pet-help-and-advice/looking-after-your-pet/kittens-cats/sphynx (accessed Feb 2025).

[r147] PDSA (undated b) *Keeping hybrid breeds as pets.* https://www.pdsa.org.uk/pet-help-and-advice/looking-after-your-pet/all-pets/hybrid-breeds (accessed Feb 2025).

[r148] Pesteanu-Somogyi LD, Radzai C and Pressler BM 2006 Prevalence of feline infectious peritonitis in specific cat breeds. Journal of Feline Medicine and Surgery 8: 1–5. 10.1016/j.jfms.2005.04.003 15994104 PMC7128820

[r149] Plitman L, Černá P, Farnworth MJ, Packer RMA and Gunn-Moore DA 2019 Motivation of owners to purchase pedigree cats, with specific focus on the acquisition of brachycephalic cats. Animals 9. 10.3390/ani9070394 PMC6680495 31252697

[r150] Polgar Z, Kinnunen M, Újváry D, Miklósi Á and Gácsi M 2016 A test of canine olfactory capacity: comparing various dog breeds and wolves in a natural detection task. PLOS One 11: e0154087. 10.1371/journal.pone.0154087 27152412 PMC4859551

[r151] Pryor PA, Hart BL, Bain MJ and Cliff KD 2001 Causes of urine marking in cats and effects of environmental management on frequency of marking. Journal of the American Veterinary Medical Association 219: 1709–1713. 10.2460/javma.2001.219.1709 11767919

[r152] Ramos D and Mills DS 2009 Human directed aggression in Brazilian domestic cats: owner reported prevalence, contexts and risk factors. Journal of Feline Medicine and Surgery 11: 835–841. 10.1016/j.jfms.2009.04.006 19577496 PMC11135496

[r153] Rand JS, Kinnaird E, Baglioni A, Blackshaw J and Priest J 2002 Acute stress hyperglycemia in cats is associated with struggling and increased concentrations of lactate and norepinephrine. Journal of Veterinary Internal Medicine 16: 123–132. 10.1111/j.1939-1676.2002.tb02343.x 11899027

[r154] Rare and Exotic Feline Registry undated *Highlander.* https://www.rareexoticfelineregistry.com/highlander/ (accessed October 2025).

[r155] Ravens PA, Xu BJ and Vogelnest LJ 2014 Feline atopic dermatitis: a retrospective study of 45 cases (2001–2012). Veterinary Dermatology 25: e28. 10.1111/vde.12109 24597491

[r156] Razlighi SS, Jahan S, and Jamshidi S 2022 Prevalence of congenital disorders, stenotic nares and malocclusion and the correlation between two disorders in brachycephalic breeds of cats referred to several private veterinary clinics in Tehran. Journal of Veterinary Research 77: 255–259. 10.22059/jvr.2022.336053.3219

[r157] Roedler FS, Pohl S and Oechtering GU 2013 How does severe brachycephaly affect dog’s lives? Results of a structured preoperative owner questionnaire. The Veterinary Journal 198: 606–610. 10.1016/j.tvjl.2013.09.009 24176279

[r158] Rowe E, Browne W, Casey R, Gruffydd-Jones T and Murray J 2015 Risk factors identified for owner-reported feline obesity at around one year of age: Dry diet and indoor lifestyle. Preventive Veterinary Medicine 121: 273–281. 10.1016/j.prevetmed.2015.07.011 26265631

[r159] Royal Canin undated *Royal Canin feline breed nutrition cat food.* https://www.royalcanin.com/ie/cats/products/feline-breed-nutrition (accessed Feb 2025).

[r160] Salgado-Caxito M, Benavides JA, Atero N, Córdova-Bürhle F, Ramos R, Fernandez M, Sapiente-Aguirre C and Mardones FO 2023 Preventive healthcare among dogs and cats in Chile is positively associated with emotional owner-companion animal bond and socioeconomic factors. Preventive Veterinary Medicine 213: 105882. 10.1016/j.prevetmed.2023.105882 36867925

[r161] Salonen M, Vapalahti K, Tiira K, Mäki-Tanila A and Lohi H 2019 Breed differences of heritable behaviour traits in cats. Scientific Reports 9. 10.1038/s41598-019-44324-x PMC6538663 31138836

[r162] Sánchez-Vizcaíno F, Noble P-JM, Jones PH, Menacere T, Buchan I, Reynolds S, Dawson S, Gaskell RM, Everitt S and Radford AD 2017 Demographics of dogs, cats, and rabbits attending veterinary practices in Great Britain as recorded in their electronic health records. BMC Veterinary Research 13: 1–13. 10.1186/s12917-017-1138-9 28693574 PMC5504643

[r163] Sandøe P, Corr S and Palmer C 2015 Companion Animal Ethics. John Wiley & Sons: London, UK.

[r164] Sandøe P, Kondrup S V, Bennett PC, Forkman B, Meyer I, Proschowsky HF, Serpell JA and Lund TB 2017a Why do people buy dogs with potential welfare problems related to extreme conformation and inherited disease? A representative study of Danish owners of four small dog breeds. PLOS One 12: e0172091. 10.1371/journal.pone.0172091 28234931 PMC5325474

[r165] Sandøe P, Nørspang AP, Forkman B, Bjørnvad CR, Kondrup SV and Lund TB 2017b The burden of domestication: A representative study of welfare in privately owned cats in Denmark. Animal Welfare 26: 1–10. 10.7120/09627286.26.1.001

[r166] Schirrer L, Marín-García PJ and Llobat L 2021 Feline polycystic kidney disease: an update. Veterinary Sciences 8: 269. 10.3390/vetsci8110269 34822642 PMC8625840

[r167] Schmidt MJ, Farke D, Staszyk C, Lang A, Büttner K, Plendl J and Kampschulte M. 2022 Closure times of neurocranial sutures and synchondroses in Persian compared to Domestic Shorthair cats. Scientific Reports 12: 573. 10.1038/s41598-022-04783-1 35022503 PMC8755779

[r168] Serpell JA 2000 Domestication and history of the cat. The Domestic Cat: The Biology of its Behaviour 2: 180–192.

[r169] Sieslack J, Farke D, Failing K, Kramer M and Schmidt MJ 2021 Correlation of brachycephaly grade with level of exophthalmos, reduced airway passages and degree of dental malalignment’in Persian cats. PLOS One 16: e0254420. 10.1371/journal.pone.0254420 34288937 PMC8294563

[r170] Stone E 2019 What’s in it for the cats?: Cat shows as serious leisure from a multispecies perspective. Leisure Studies 38: 381–393. 10.1080/02614367.2019.1572776

[r171] Ström Holst B, Axnér E, Öhlund M, Möller L and Egenvall A 2017 Dystocia in the cat evaluated using an insurance database. Journal of Feline Medicine and Surgery 19: 42–47. 10.1177/1098612X15600482 26297020 PMC10816746

[r172] Ström Holst B and Frössling J 2009 The Swedish breeding cat: population description, infectious diseases and reproductive performance evaluated by a questionnaire. Journal of Feline Medicine and Surgery 11: 793–802. 10.1016/j.jfms.2009.01.008 19254857 PMC7129517

[r173] Struck AK, Braun M, Detering KA, Dziallas P, Neßler J, Fehr M, Metzger J and Distl O 2020 A structural UGDH variant associated with standard Munchkin cats. BMC Genetics 21. 10.1186/s12863-020-00875-x PMC7325026 32605545

[r174] Takanosu M and Hattori Y 2020 Osteochondrodysplasia in scottish fold cross-breed cats. Journal of Veterinary Medical Science 82: 1769–1772. 10.1292/jvms.20-0299 33162427 PMC7804039

[r175] Tan SML, Stellato AC and Niel L 2020 Uncontrolled outdoor access for cats: An assessment of risks and benefits. Animals 10: 258. 10.3390/ani10020258 32041155 PMC7070728

[r176] Tasker S, Addie DD, Egberink H, Hofmann-Lehmann R, Hosie MJ, Truyen U, Belák S, Boucraut-Baralon C, Frymus T, Lloret A, Marsilio F, Pennisi MG, Thiry E, Möstl K and Hartmann K 2023 Feline Infectious Peritonitis: European Advisory Board on Cat Diseases Guidelines. Viruses 15. 10.3390/v15091847 PMC10535984 37766254

[r177] Tateo A, Zappaterra M, Covella A and Padalino B 2021 Factors influencing stress and fear-related behaviour of cats during veterinary examinations. Italian Journal of Animal Science 20: 46–58. 10.1080/1828051x.2020.1870175

[r178] Tatlock S, Gober M, Williamson N and Arbuckle R 2017 Development and preliminary psychometric evaluation of an owner-completed measure of feline quality of life. Veterinary Journal 228: 22–32. 10.1016/j.tvjl.2017.10.005 29153104

[r179] Taylor PM and Robertson SA 2004 Pain management in cats—past, present and future. Part 1. The cat is unique. Journal of Feline Medicine and Surgery 6: 313–320. 10.1016/j.jfms.2003.10.003 15363763 PMC10822206

[r180] Taylor S, Tasker S, Gunn-Moore D, Barker E and Sorrell S 2024 An update on treatment of FIP using antiviral drugs in 2024: growing experience but more to learn. https://icatcare.org/app/uploads/2024/11/FIP-VET-update-November-2024.pdf (accessed Jan 2025).

[r181] TICA (undated a) *Browse All Breeds.* https://tica.org/ticas-breeds/non-championship-breeds/ (accessed Feb 2025).

[r182] TICA (undated b) *Non-Championship breeds.* https://tica.org/ticas-breeds/non-championship-breeds/ (accessed Feb 2025).

[r183] TICA (undated c) *Minuet.* https://tica.org/breed/minuet/ (accessed Feb 2025).

[r184] TICA (undated d) *Highlander.* https://tica.org/breed/highlander/ (accessed Oct 2025).

[r185] The Cat Group 2024 *The Cat Group Position Statement on ‘Bully’ and ‘Dwelf’ Cats.* https://bvna.org.uk/blog/the-cat-group-position-statement-on-bully-and-dwelf-cats/ (accessed October 2025).

[r186] Trehiou‐Sechi E, Tissier R, Gouni V, Misbach C, Petit AMP, Balouka D, Carlos Sampedrano C, Castaignet M, Pouchelon J and Chetboul V 2012 Comparative echocardiographic and clinical features of hypertrophic cardiomyopathy in 5 breeds of cats: a retrospective analysis of 344 cases (2001–2011). Journal of Veterinary Internal Medicine 26: 532–541. 10.1111/j.1939-1676.2012.00906.x 22443341

[r187] Tsai H-Y, Chueh L-L, Lin C-N and Su B-L 2011 Clinicopathological findings and disease staging of feline infectious peritonitis: 51 cases from 2003 to 2009 in Taiwan. Journal of Feline Medicine and Surgery 13: 74–80. 10.1016/j.jfms.2010.09.014 21216644 PMC7129202

[r188] Ukawa H, Kida A, Ataka K, Horie R, and Matsumoto Y 2024 Widespread genetic testing control inherited polycystic kidney disease in cats. *bioRxiv.* 10.1101/2024.12.15.628535 PMC13520345 42316006

[r189] UK Brachycephalic Working Group 2022 *Innate health in dogs.* https://www.ukbwg.org.uk/wp-content/uploads/2022/05/220512-BWG-Innate-health-in-dog-populations.pdf (accessed Feb 2025).

[r190] UK Parliament 2022 *Dangerous Wild Animal Act 1976.* https://www.legislation.gov.uk/ukpga/1976/38 (accessed Feb 2025).

[r191] van Bree FPJ, Bokken GCAM, Mineur R, Franssen F, Opsteegh M, van der Giessen JWB, Lipman LJA and Overgaauw PAM 2018 Zoonotic bacteria and parasites found in raw meat‐based diets for cats and dogs. Veterinary Record 182: 50. 10.1136/vr.104535 29326391

[r192] van Hagen MAE 2019 *Breeding short-muzzled dogs.* https://www.uu.nl/sites/default/files/eng_breeding_short-muzzled_dogs_in_the_netherlands_expertisecentre_genetics_of_companionanimals_2019_translation_from_dutch.pdf (accessed Jan 2025).

[r193] Vapalahti K, Neittaanmäki H, Lohi H and Virtala A-M 2024 A large case-control study indicates a breed-specific predisposition to feline tooth resorption. The Veterinary Journal 305: 106133. 10.1016/j.tvjl.2024.106133 38740176

[r194] Velie BD, Milden T, Miller H and Haase B 2023 An estimation of osteochondrodysplasia prevalence in Australian Scottish Fold cats: a retrospective study using VetCompass Data. BMC Veterinary Research 19: 252. 10.1186/s12917-023-03811-0 38031079 PMC10685627

[r195] Vigne JD, Guilaine J, Debue K, Haye L and Gérard P 2004 Early Taming of the Cat in Cyprus. Science 304: 259. 10.1126/science.1095335 15073370

[r196] Walker C, Vierck CJ and Ritz LA 1998 Balance in the cat: role of the tail and effects of sacrocaudal transection. Behavioural Brain Research 91: 41–47. 10.1016/s0166-4328(97)00101-0 9578438

[r197] Wall M, Cave NJ and Vallee E 2019 Owner and cat-related risk factors for feline overweight or obesity. Frontiers in veterinary Science 6: 266. 10.3389/fvets.2019.00266 31482097 PMC6709657

[r198] Wassink-van der Schot AA, Day C, Morton JM, Rand J and Phillips CJC 2016 Risk factors for behavior problems in cats presented to an Australian companion animal behavior clinic. Journal of Veterinary Behavior: Clinical Applications and Research 14: 34–40. 10.1016/j.jveb.2016.06.010

[r199] Wastlhuber J 1991 History of domestic cats and cat breeds. In: Pedersen NC (ed) Feline Husbandry pp. 1–59. American Veterinary Publications: Goleta, CA, USA.

[r200] Wilhelmy J, Serpell J, Brown D and Siracusa C 2016 Behavioral associations with breed, coat type, and eye color in single-breed cats. Journal of Veterinary Behavior 13: 80–87. 10.1016/j.jveb.2016.03.009

[r201] Winzar H 2015 The ecological fallacy: How to spot one and tips on how to use one to your advantage. Australasian Marketing Journal 23: 86–92. 10.1016/j.ausmj.2014.12.002

[r202] Worboys M, Strange JM and Pemberton N 2018 *The Invention of the Modern Dog: Breed and Blood in Victorian Britain*, *First Edition*. Johns Hopkins University Press: Baltimore, Maryland, USA.

[r203] Worthing KA, Wigney DI, Dhand NK, Fawcett A, McDonagh P, Malik R and Norris JM 2012 Risk factors for feline infectious peritonitis in Australian cats. Journal of Feline Medicine and Surgery 14: 405–412. 10.1177/1098612X12441875 22398460 PMC10822597

[r204] WSAVA undated *Hereditary Disease Committee.* https://wsava.org/committees/hereditary-disease-committee/ (accessed Oct 2024).

