# Supplementary material for: A framework to refocus the conversation around the welfare of UK purebred cats
Source: Anim Welf. 2025 Nov 28;34:e76. doi: 10.1017/awf.2025.10051 (PMC12722057; doi:10.1017/awf.2025.10051)
Supplement: Roberts et al. supplementary material [file S0962728625100511sup001.pdf]

A framework to refocus the conversation around the welfare of UK purebred cats: Supplementary material

Claire Roberts https://orcid.org/0000-0002-0790-0664 [1], Rae Foreman-Worsley [1], Dan G O'Neill [2] and Jennifer McDonald [1]

[1] Feline Welfare and Operations Directorate, Cats Protection, National Cat Centre, Haywards Heath, UK

[2] Pathobiology and Population Sciences, The Royal Veterinary College, Hawkshead Lane, North Mymms, Hatfield, Herts AL9 7TA, UK

Author for correspondence: Claire Roberts, email: claire.roberts@cats.org.uk

Table S1. Cat breeds accepted by UK registration bodies according to our current knowledge, any extreme features and whether they have identified health or behavioural predispositions (as listed in Gough *et al.* 2018).

	UK registration			Extreme features							Identified predispositions	
**Breed**	**TICA**	**GCCF**	**Felis Britannica**	**Skull shape**	**No or shortened tail**	**Curled/folded Ears**	**Hair coat divergence**	**Dwarfism**	**Other**	**Hybrid cat**	**Disease**	**Behavioural**
Abyssinian	Yes	Yes	Yes	Dolichocephalic	No	No	No	No	No	No	Yes	Yes
Asian*	No	Yes	No	Mesocephalic	No	No	No	No	No	No	Not identified	Not identified
American Bobtail*	Yes	Grade B‡	No	Mesocephalic	Yes	No	No	No	No	No	Not identified	Not identified
American Curl*	Yes	Grade B‡	Yes	Mesocephalic	No	Yes	No	No	No	No	Not identified	Not identified
American Shorthair	Yes	Grade A‡	No	Brachycephalic	No	No	No	No	No	No	Not identified	Not identified
American Wirehair	Yes	Grade A‡	No	Brachyceplalic	No	No	No	No	No	No	Not identified	Not identified
Aphrodite	Yes	No	No	Mesocephalic	No	No	No	No	No	No	Not identified	Not identified
Australian mist	Yes	Yes	No	Mesocephalic derived from burmese	No	No	No	No	No	No	Yes	Not identified
Balinese	Yes	Yes	Yes	Dolichocephalic	No	No	No	No	No	No	Yes	Not identified
Bengal*	Yes	Yes (shorthair only)	Yes	Dolichocephalic	No	No	No	No	No	Yes: Asian leopard cat *Prionailurus bengalensis*	Yes	Yes
Birman	Yes	Yes	Yes as Sacred Birman	Mesocephalic	No	No	No	No	No	No	Yes	Yes
Birmilla	No	No	Yes	Brachycephalic	No	No	No	No	No	No	Not identified	Not identified
Bombay	Yes	No	Preliminary	Brachycephalic	No	No	No	No	No	No	Not identified	Not identified
British*	Yes	Yes	Yes	Brachycephalic	No	No	No	No	No	No	Yes (shorthair)	Yes
Burmese	Yes	Yes	Yes	Brachycephalic	No	No	No	No	No	No	Yes	Yes
Burmilla*	Yes	No	Yes	Mesocephalic	No	No	No	No	No	No	Not identified	Not identified
Chartreux	Yes	Yes	Yes	Mesocephalic	No	No	No	No	No	No	Yes	Not identified

Chausie	Yes	No	No	Mesocephalic	No	No	No	No	No	Yes: Jungle cat *Felis chaus*	Not identified	Not identified
Cornish rex	Yes	Yes	Yes	Mesocephalic	No	No	Curly coat	No	No	No	Yes	Yes
Cymric	Yes	As longhaired variant of Manx	yes	Mesocephalic	Yes	No	No	No	No	No	Not identified	Not identified
Devon rex	Yes	Yes	Yes	Dolichocephalic	No	No	Curly coat	No	No	No	Yes	Yes
Donskoy	Yes	No	Yes (as Don sphynx)	Dolichocephalic	No	No	No	No	No	No	Not identified	Not identified
Egyptian Mau	Yes	Yes	Yes	Dolichocephalic	No	No	No	No	No	No	Yes	Not identified
European shorthair	Registration only†	No	Yes	Brachycephalic	No	No	No	No	No	No	Yes	Not identified
Exotic shorthair	Yes	Yes	Yes	Brachycephalic	No	No	No	No	No	No	Yes	Not identified
German Rex	No	No	Yes	Mesocephalic	No	No	Curly coat	No	No	No	Not identified	Not identified
Havana	Yes	No	No	Mesocephalic	No	No	No	No	No	No	Yes	Not identified
Highlander*	Advanced new breed†	No	No	Mesocephalic	Yes	Yes	No	No	No	Unknown§	Not identified	Not identified
Himalayan	Yes	No	No	Brachycephalic	No	No	No	No	No	No	Yes	Not identified
Japanese Bobtail*	Yes	Grade B‡	Yes (shorthair)	Mesocephalic	Yes	No	No	No	No	No	Yes	Not identified
Khaomanee	Yes	No	No	Mesocephalic	No	No	No	No	No	No	Not identified	Not identified
Korat	Yes	Yes	Yes	Mesocephalic	Yes	No	No	No	No	No	Yes	Yes
Kurilian Bobtail*	Yes	Grade B‡	Yes	Mesocephalic	No	No	No	No	No	No	Not identified	Not identified
LaPerm*	Yes	Yes	Yes	Mesocephalic	No	No	Curly coat	No	No	No	Yes	Not identified
Lykoi*	Yes	Yes	Preliminary	Mesocephalic	No	No	Reduced coat	No	No	No	Not identified	Not identified
Maine Coon	Yes	Yes	Yes	Mesocephalic	No	No	No	No	No	No	Yes	Yes
Maine Coon Polydactyl	Yes	No	No	Mesocephalic	No	No	No	No	Yes	No	Not identified	Not identified

Manx	Yes	Yes	Yes	Mesocephalic	Yes	No	No	No	No	No	Yes	Not identified
Minuet*	Yes	No	No	Brachycephalic	No	No	No	Yes	No	No	Not identified	Not identified
Munchkin*	Yes	No	No	Mesocephalic	No	No	No	Yes	No	No	Not identified	Not identified
Nebelung	Yes	Yes	No	Mesocephalic	No	No	No	No	No	No	Not identified	Not identified
Neva Masquerade	No	No	Yes	Mesocephalic	No	No	No	No	No	No	Not identified	Not identified
Norwegian Forest	Yes	Yes	Yes	Mesocephalic	No	No	No	No	No	No	Yes	Not identified
Ocicat	Yes	Yes	Yes	Mesocephalic	No	No	No	No	No	No	Yes	Not identified
Oriental*	Yes	Yes	Yes	Dolichocephalic	No	No	No	No	No	No	Yes (shorthair)	Yes
Persian	Yes	Yes	Yes	Brachycephalic	No	No	No	No	No	No	Yes	Yes
Peterbald	Yes	No	Yes	Mesocephalic	No	No	Hairless/thin coat	No	No	No	Not identified	Not identified
Pixiebob*	Yes	No	No	Mesocephalic	No	No	No	No	Prone to polydactyly	No	Not identified	Not identified
RagaMuffin	No	Yes	No	Mesocephalic	No	No	No	No	No	No	Not identified	Not identified
Ragdoll	Yes	Yes	Yes	Mesocephalic	No	No	No	No	No	No	Yes	Yes
Russian Blue	Yes	Yes (as Russian)	Yes	Mesocephalic	No	No	No	No	No	No	Yes	Yes
Savannah	Yes	No	No	Mesocephalic	No	No	No	No	No	Yes: African Serval *Leptailurus serval*	Yes	Not identified
Scottish Fold*	Yes	No	No	Brachycephalic	No	Yes	No	No	No	No	Yes	Not identified
Scottish Straight*	Yes	No	No	Brachycephalic	No	No	No	No	No	No	Not identified	Not identified
Selkirk Rex*	Yes	Yes	Yes	Mesocephalic	No	No	Curly coat	No	No	No	Not identified	Not identified
Serengeti	Advanced new Breed[†]	Grade A[‡]	No	Mesocephalic	No	No	No	No	No	No	Not identified	Not identified
Siamese	Yes	Yes	Yes	Dolichocephalic	No	No	No	No	No	No	Yes	Yes
Siberian	Yes	Yes	Yes	Mesocephalic	No	No	No	No	No	No	Yes	Not identified

Singpura	Yes	Yes	Yes	Mesocephalic	No	No	No	No	No	No	Yes	Not identified
Snowshoe	Yes	Yes	Yes	Mesocephalic	No	No	No	No	No	No	Not identified	Not identified
Sokoke	No	Yes	Yes	Mesocephalic	No	No	No	No	No	No	Not identified	Not identified
Somali	Yes	Yes	Yes	Mesocephalic	No	No	No	No	No	No	Yes	Not identified
Sphynx	Yes	Yes	Yes	Dolicocephali c	No	No	Hairless/th in coat	No	No	No	Yes	Not identified
Suffolk	No	Yes	No	Mesocephalic	No	No	No	No	No	No	Not identified	Not identified
Tennesee Rex	Yes	Grade A‡	No	Mesocephalic	No	No	Curly coat	No	No	No	Not identified	Not identified
Thai	Yes	Yes	Yes	Mesocephalic	No	No	No	No	No	No	Not identified	Not identified
Tonkinese	Yes	Yes	No	Mesocephalic	No	No	No	No	No	No	Yes	Yes
Toybob	Prelimi nary new breed†	Grade B‡	No	Mesocephalic	Yes	No	No	No	No	No	Not identified	Not identified
Toyger	Yes	Yes	No	Mesocephalic	No	No	No	No	No	Yes: derived from Bengal	Not identified	Not identified
Turkish Angora	Yes	No	Yes	Mesocephalic	No	No	No	No	No	No	None listed	Not identified
Turkish Van	Yes	Yes	Yes	Mesocephalic	No	No	No	No	No	No	None listed	Yes

*Short and longhair types

†Registration only: can be registered but not yet shown; Preliminary and advanced new breeds: can be shown but cannot yet win titles (new breeds progress through these stages)

‡ Grade A: Probable acceptance; Grade B: acceptance depends on genetic and veterinary information

§TICA reports Highlander is derived from the “domestic gene pool” whereas the Rare and Exotic Feline Registry report the breed to be derived from the Jungle Curl and Desert Lynx

Table S2. Hybrid pure cat breeds not to our knowledge currently accepted by any UK cat registration bodies.

Breed	Wildcat(s) hybridised
Bristol	Margay *(Leopardus wiedii)*
Caracat	Caracal *(Caracal caracal)*
Cheetoh	Bengal and Ocicat breeds
Jaguarundi Curl	Jaguarundi *(Puma yagouaroundi)*
Jambi	Fishing cat *(Prionailurus viverrinus)*
Marguerite	Sandcat *(Felis margarita)*
Marlot	Ocelot *(Leopardus pardalis)* and Margay *(Leopardus weidii)*
Punjabi	Indian desert cat *(Felis silvestris ornata)*
Safari cat	Geoffory's cat *(Leopardus geoffroyi)*

Table S3. Non-hybrid pure breeds not to our knowledge currently accepted by any UK cat registration bodies. Note: these breeds may have extreme features that are not obvious in online images.

Breed	Breed origin	Obvious extreme features
Aegean	Natural breed	None
American Ringtail	Experimental: From domestic cat with a tail mutation	Curled tail
Arabian Mau	Natural breed	None
Bahraini Dilmun Cat/Delmun Cat	Natural breed	None
Bambino	Experimental: Sphynx/Munchkin	Dwarf, hairless
Brazilian*	Experimental: Origins unclear	None
California Spangled	Experimental: Various breeds	None
Exotic Long Hair	Experimental: Persian descent	brachycephalic
Foreign White	Experimental: White Siamese variant	Dolichocephalic
Javanese	Experimental: Variant of Balinese	Dolichocephalic
Keetso	Experimental: Origins unclear	Polydactyl
Minskin	Experimental: Various brees including Munchkin, Sphynx	Dwarf, hairless
Ragamese	Experimental: Ragdoll/Siamese	None
Seychellois*	Experimental: Siamese/Persian/Oriental	Dolichocephalic
Tibetan	Experimental: Balinese/Tonkinese	None
XL Bully Cat	Experimental: Munchkin/Sphynx	Dwarf, hairless
York Chocolate	Experimental: Siamese descent	None

*Short and long hair variants.
